# Rhythm control benefits left ventricular function compared with rate control in patients with atrial fibrillation: A computational study

**DOI:** 10.1016/j.hroo.2025.04.014

**Published:** 2025-05-09

**Authors:** Rosie K. Barrows, Christoph M. Augustin, Matthias A.F. Gsell, Caroline H. Roney, José A. Solís Lemus, Hao Xu, Alistair A. Young, Ronak Rajani, John Whitaker, Edward J. Vigmond, Martin J. Bishop, Gernot Plank, Marina Strocchi, Steven A. Niederer

**Affiliations:** 1 School of Biomedical Engineering and Imaging Sciences, King’s College London, London, United Kingdom; 2 National Heart and Lung Institute, Imperial College London, London, United Kingdom; 3 Gottfried Schatz Research Center, Division of Medical Physics and Biophysics, Medical University of Graz, Graz, Austria; 4 BioTechMed-Graz, Graz University of Technology, Graz, Austria; 5 School of Engineering and Materials Science, Queen Mary University of London, London, United Kingdom; 6 Liryc Heart Rhythm Disease Institute, Fondation Bordeaux University, Pessac-Bordeaux, France; 7 CNRS, Bordeaux INP, IMB, University of Bordeaux, Bordeaux, France; 8 The Alan Turing Institute, London, United Kingdom

**Keywords:** Atrial fibrillation, Rate control, Rhythm control, Electromechanics modeling, Cardiac modeling, In-silico trial

## Abstract

**Background:**

Atrial fibrillation (AF) alters heart rate, rhythm regularity, and atrial contraction, which may contribute to an increased risk of heart failure. Although rate and rhythm control target different aspects of these disturbances, their specific effects on left ventricular (LV) function remain unclear.

**Objective:**

The purpose of this study was to predict the independent and combined contribution of heart rate, rhythm regularity, and atrial contraction to LV function in patients with AF.

**Methods:**

We predicted LV ejection fraction (EF) and stroke volume (SV) in 10 whole-heart patient-specific models of patients with AF while varying heart rate, rhythm regularity, and effectiveness of atrial contraction.

AF was modeled as a fast, irregular heart rate with no atrial contraction. Pharmacologic and paced rate control were modeled as a slow irregular and regular heart rate without atrial contraction, respectively, whereas rhythm control had a slow, regular heart rate with atrial contraction.

**Results:**

Rhythm control resulted in a greater improvement than pharmacological rate control in LVEF compared with AF (+5.1% ± 0.4% vs +2.8% ± 0.3%, *P* < .01). Paced rate control was equivalent to pharmacologic rate control in terms of LVEF (+2.6% ± 0.4% vs +2.8% ± 0.3%). Atrial contraction did not improve ventricular function in the presence of an irregular heart rate (pharmacologic rate: +2.8% ± 0.3% vs rhythm with irregular heart rate: +2.7% ± 0.3%).

**Conclusion:**

Rhythm control provides superior improvements in LV function compared with rate control. However, restoring sinus rhythm may yield limited benefits to LV function when atrial contraction is ineffective or when heart rate is irregular.

## Graphical abstract

**Figure:**
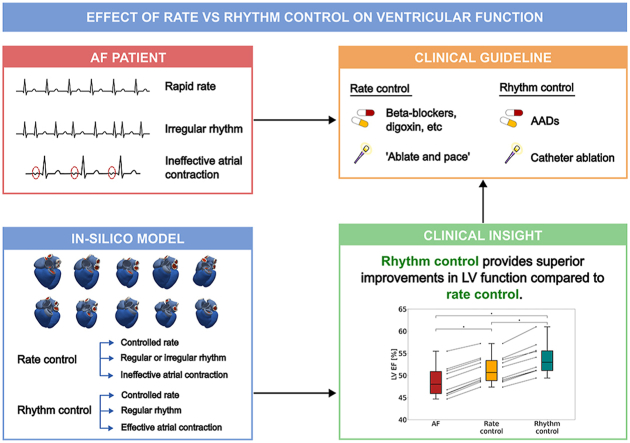

**Key Findings**

▪ Physiological ventricular rate, regular rhythm, and effective atrial contraction, all significantly contribute to healthy left ventricular function, as measured by stroke volume and ejection fraction.
▪ Rhythm control achieves superior improvement in left ventricular function than rate control in patients with atrial fibrillation (AF).
▪ The additional benefits of rhythm control are likely limited to patients in whom both effective atrial contraction and a regular rhythm can be restored.
▪ In patients with significant atrial fibrosis, scarring from ablation, or persistent ectopic activity, pharmacologic rate control may offer a comparable alternative to rhythm control for AF management.

## Introduction

Atrial fibrillation (AF), characterized by an irregular heart rate with loss of atrial contraction,^1^ is the most common cardiac arrhythmia and has a rapidly increasing prevalence.^2^ Onset of AF is often accompanied by an accelerated ventricular rate and, in some cases, deterioration of left ventricular (LV) function. Management of AF may include attempts to control the ventricular rate (rate control) or to restore sinus rhythm (SR) (rhythm control). Rate control can be achieved by medication or atrioventricular-junction ablation and pacing. Data from the early 2000s indicated that rate control was not inferior to rhythm control for the prevention of death and morbidity because of cardiovascular causes.^3^, ^4^, ^5^, ^6^, ^7^ However, more recent studies have found that early rhythm control is associated with a lower risk of adverse cardiovascular outcomes^8^ and improved exercise tolerance^5^^,^^7^ than is usual care. It is now acknowledged that in patients with established heart failure, restoration and maintenance of SR are associated with a mortality benefit,^9^ and more broadly, that maintenance of SR is associated with reduced onset of heart failure and other adverse cardiovascular outcomes.^10^

Differences in outcome with rate and rhythm control may in part be due to improved LV function with rhythm control compared with rate control,^11^ given a reduction in LV ejection fraction (EF) in patients with AF can cause or worsen heart failure.^12^ Previous simulation^13^ and clinical studies^14^ have identified heart rate irregularity as an important determinant of LV function in AF. However, the interaction of an irregular heart rate with a rapid heart rate and lack of atrial contraction in AF was not explored. How these distinct mechanisms interact to give rise to changes in LV function with rate and rhythm control strategies in AF management remains unexplored.

Computational models of the heart offer a noninvasive tool to quantify response to treatment in patients with cardiovascular diseases. These models enable the comparison of therapeutic strategies without the confounding effects of adverse drug reactions, patient noncompliance, or switching of therapy. Furthermore, AF clinical trials often do not test the same treatment in the same patient. Although cross-over trials offer some ability to address this, they are limited in the context of AF because treatments may include an ablation, which complicates repeated interventions. As a result, making direct, pairwise comparisons of treatments become challenging. Moreover, modeling studies allow treatment strategies to be compared directly and flexibly, in contrast to some clinical trials that evaluate complex combinations, such as pharmacologic and surgical rate control vs pharmacologic and surgical rhythm control. Cardiac modeling has already provided insight into the response to cardiac resynchronization therapy, AF, and ventricular tachycardia ablation.^15^, ^16^, ^17^ However, these models either used a simplified and generalized heart anatomy or failed to capture the mechanism of interaction between atria and ventricles because they considered only 1 or 2 chambers. In contrast, whole-heart patient-specific models can simulate these mechanisms,^18^, ^19^, ^20^ allowing them to investigate interchamber interactions in AF therapies.

In this study, we used patient-specific whole-heart electromechanics modeling to predict changes in LVEF and stroke volume (SV) achieved with rate or rhythm control by varying heart rate, rhythm regularity, and effectiveness of atrial contraction in a cohort of models of patients with AF. Combinations of these factors were then used to simulate pharmacologic or paced rate control, and rhythm control.

## Methods

### Patient cohort

**Figure 1:**
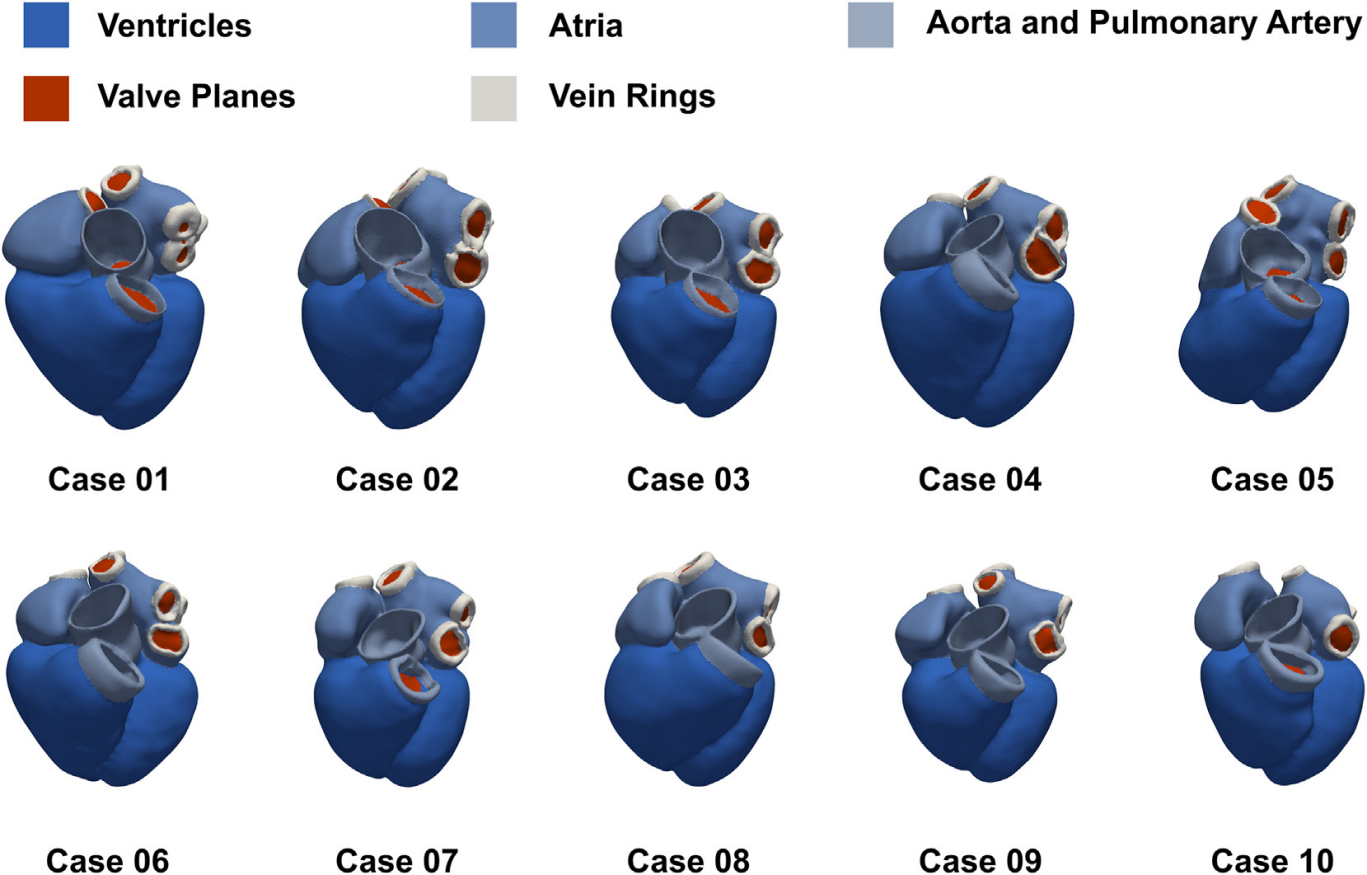
Patient-specific meshes. Each model was generated from a computed tomography scan of a patient with atrial fibrillation. The cohort of models represents male and female patients with a range of ages, body mass indices, and ventricular volumes.

We used imaging data from 10 patients with AF (Table 1). Patients underwent electrocardiography (ECG)-gated computed tomography (CT). The imaging data were collected as part of a prospective study that received ethical approval from the Westminster Research Ethics Committee (reference No. 15/LO/1803). The study conformed with the Declaration of Helsinki, and all participants provided written, informed consent. We automatically segmented the end-diastolic CT images and generated 10 patient-specific 4-chamber meshes using an existing pipeline.^21^, ^22^, ^23^ The models include ventricular and atrial fibers and representations of the Bachmann bundle and a fast endocardial conduction layer.^24^, ^25^, ^26^ The cohort of models is presented in Figure 1.

**Table 1:** Demographic data of patients with AF

Demographic	Value
Age [y]	63 ± 10
Sex [% male]	60
BMI [kg/m^2^]	28 ± 2.8
AF classification	Paroxysmal
EDV_LV_ [mL]	117 ± 30
AAR [%]	60
(Flecainide)	(40)
(Sotalol)	(20)
(Amiodarone)	(10)
BB/CCB [%]	50
(Bisoprolol)	(40)
(Diltiazem)	(10)

AAR = antiarrhythmic therapy; AF = atrial fibrillation; BB = β blocker; BMI = body mass index; CCB = calcium channel blocker; EDV_LV_ = left ventricular end-diastolic volume.

### Electromechanical simulations

The model captures activation propagation at the tissue scale,^27^ coupled with models of human atrial and ventricular cellular physiology.^28^^,^^29^ The atrial cell model was modified to include the effect of calcium/calmodulin-dependent protein kinase II to appropriately capture the rate-dependency of the calcium dynamics (Supplemental Materials 1 and 2). Conduction velocity in the fiber and transverse direction was calibrated to achieve physiological activation times in the atria and ventricles (Supplemental Material 3).^30^^,^^31^

A model with atrial and ventricular variants of tension development was coupled with the atrial and ventricular electrophysiological cell models through the calcium transient.^32^ The passive material properties of the myocardium were represented using a model that accounts for its anisotropic elastic behavior and stress-strain relationships.^33^ All other structures were modeled as elastic materials that exhibit nonlinear deformation characteristics (Supplemental Material 4). The effect of the pericardium was included by applying spatially varying normal springs to the epicardium.^21^ A closed-loop model of the circulatory system was used to simulate physiological preload and afterload on all 4 chambers,^34^ and the effect of including the baroreflex was investigated in Supplemental Material 5. The Cardiac Arrhythmia Research Package was used for all electromechanical simulations.^35^^,^^36^

### Model calibration

Each patient model was calibrated to achieve physiological aortic systolic pressure (90–140 mm Hg), aortic diastolic pressure (60–90 mm Hg),^37^^,^^38^ LVEF (> 50%),^39^ and the image derived end-diastolic volume (EDV). Details of the parameter-fitting process are provided in Supplemental Material 6.

### AF factorial study design

To quantify the relative contribution of heart rate, regularity, and atrial contraction to LV function, a 2-level factorial study design was selected. Table 2 lists the combinations of these factors and gives their clinical significance, when appropriate.

**Table 2:** Factorial study design with clinical interpretations

Rate	Regularity	Atrial contraction	Clinical interpretation
Physiological	Regular	Yes	Successfully restored sinus rhythm
Physiological	Regular	No	Paced rate-controlled AF
Physiological	Irregular	Yes	Sinus rhythm with irregularity
Physiological	Irregular	No	Pharmacologically rate-controlled AF
Rapid	Regular	Yes	Exercise
Rapid	Regular	No	-
Rapid	Irregular	Yes	-
Rapid	Irregular	No	Rapidly conducted AF

AF = atrial fibrillation.

Successfully restored SR (henceforth referred to as rhythm control) was modeled as a regular and physiological heart rate with effective atrial contraction. AF managed with atrioventricular-junction (or atrioventricular node) ablation and ventricular pacing (referred to as paced rate control) was modeled as a regular and physiological heart rate without effective atrial contraction. Simulations of paced rate-controlled AF assume physiological ventricular activation although it should be noted that complete physiological activation may not be achieved with pacing. AF that has been managed with drugs to slow the ventricular rate (referred to as pharmacologic rate control) was modeled as an irregular and physiological heart rate without effective atrial contraction. AF treated with a rhythm control strategy but in which a degree of irregularity remained (referred to as SR with irregularity) was modeled as an irregular and physiological heart rate with effective atrial contraction. Finally, rapidly conducted AF (referred to as untreated AF) was modeled as an irregular and rapid heart rate without effective atrial contraction. In each case, ineffective atrial contraction was modeled by preventing tension development in the atrial cell model. Simulations with regular rhythms were run for 5 beats to achieve a hemodynamically steady-state solution, and the final beat was analyzed. Simulations with irregular rhythm were run with an initial 3 irregular RR-intervals to provide a quasi-steady state, followed by a further 9 irregular R-R intervals. Irregular R-R intervals were sampled from distributions of R-R intervals generated from AF ECGs (Supplemental Material 7). The average outputs of the final 9 beats were used for analysis. The average R-R interval for each AF ECG was used to provide the basic cycle length for the physiological and rapid heart rate simulations (61 and 94 beats per minute [bpm], respectively). For each scenario, we quantified LV function by computing LVEF and LVSV.

### Statistical analysis

We used paired *t* tests to compare different scenarios, with a significance level of *P* < .01. We used Bonferroni correction to correct for multiple comparisons.

## Results

In this section, the most clinically relevant findings are discussed. Results from the full factorial investigation are available in Supplemental Material 8; data on ventricular and systolic/diastolic pressures are available in Supplemental Material 9, and results stratified by sex are provided in Supplemental Material 10.

### Rate, regular rhythm, and atrial contraction all contribute significantly to LV function

**Figure 2:**
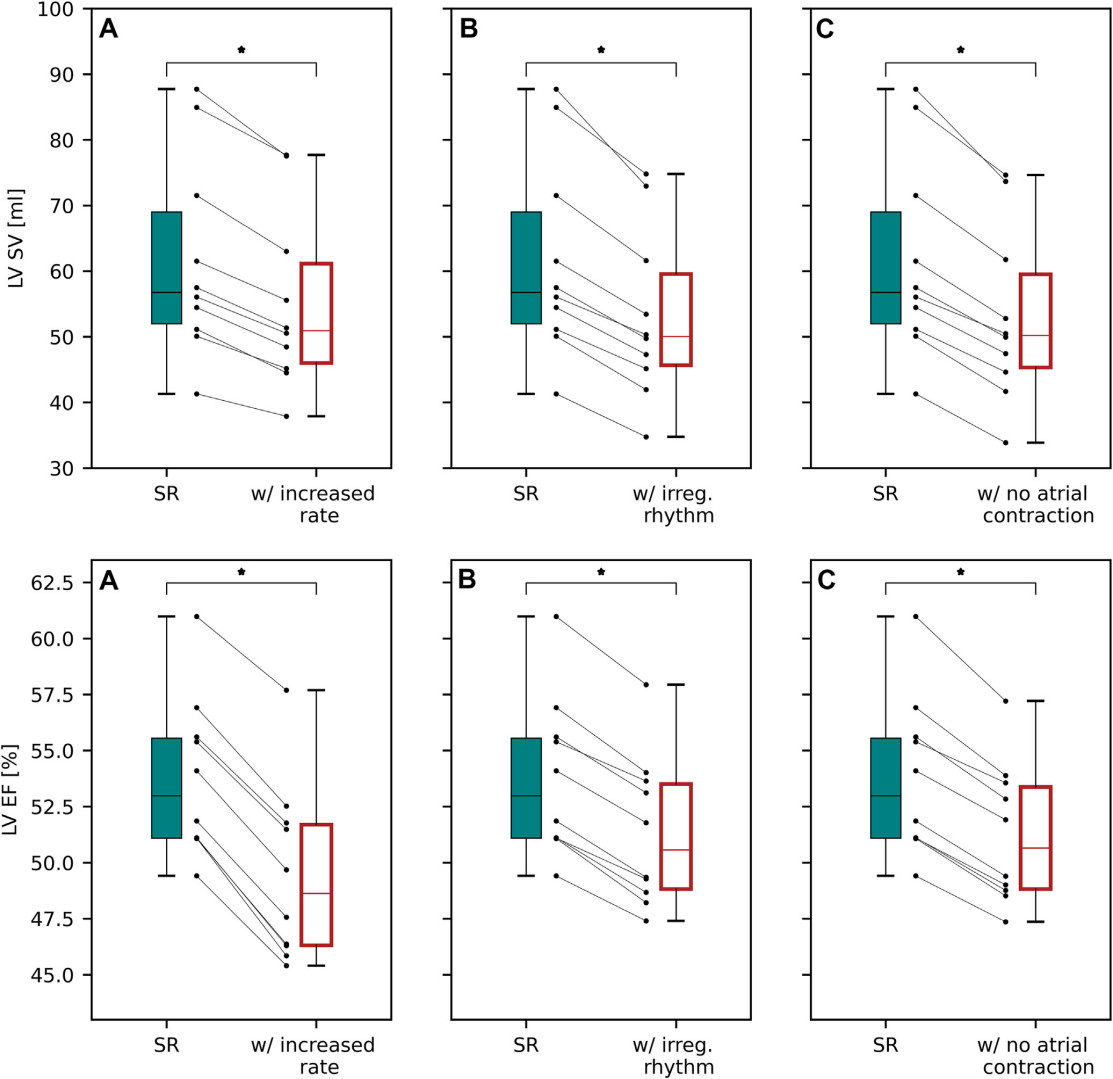
Contribution of heart rate, regularity, and atrial contraction to left ventricular function: Box plots of the LVSV (*top*) and LVEF (*bottom*) for simulated SR compared with simulations with **A:** rapid, regular rhythm with effective atrial contraction; **B:** physiological, irregular rhythm with effective atrial contraction; and **C:** physiological, regular rhythm without effective atrial contraction. Scatter points represent the results for each heart in the cohort. irreg. = irregular; LVEF = left ventricular ejection fraction; LVSV = left ventricular stroke volume; SR = sinus rhythm.

The calibrated virtual cohort had significantly impaired LV function during untreated AF compared with SR (LVEF 53.8% ± 3.5% vs 48.6% ± 3.5%, LVSV 61.6 ± 15.2 mL vs 50.0 ± 12.2 mL). To test the independent contribution of rate, regularity, and atrial contraction to LV function, we simulated SR with increased heart rate, an irregular rhythm, or with no effective atrial contraction (Figure 2). LVSV decreased by 10.4%, 13.7%, and 14.0% with an increased heart rate, an irregular heart rate, and no atrial contraction, respectively. LVEF decreased from 53.8% ± 3.5% in SR to 49.5% ± 3.9% (*P* < .01), 51.3% ± 3.3% (*P* < .01) and 51.2% ± 3.1% (*P* < .01) with an increased heart rate, an irregular heart rate, and no atrial contraction, respectively. All 3 factors affected LV function, with elevated heart rate having a greater effect on LVEF than the loss of atrial contraction or presence of an irregular rhythm.

### Rhythm control confers a significant benefit compared with rate control

**Figure 3:**
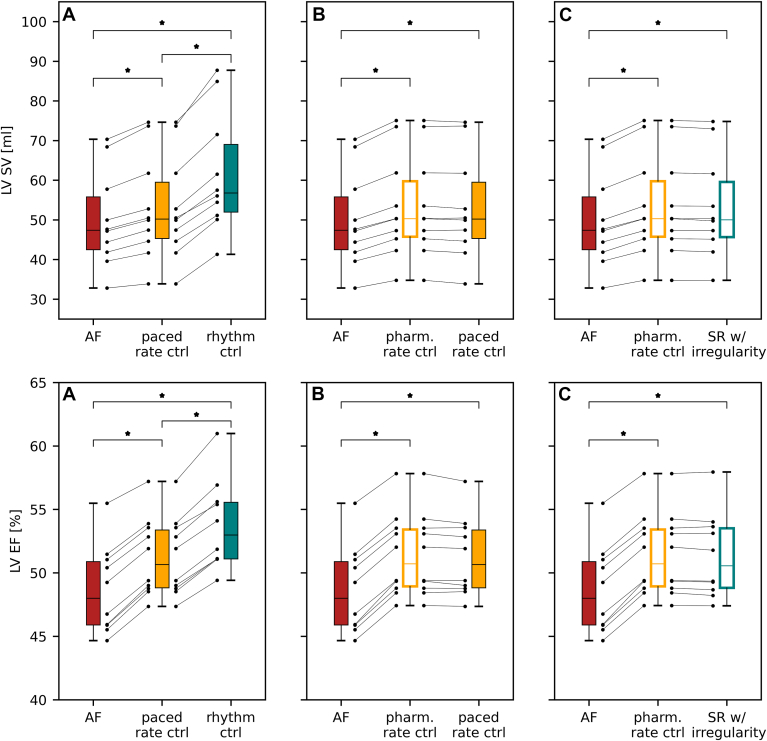
The effect of different AF management strategies on LV function: Box plots of the LVSV and LVEF for untreated AF compared with simulations with **A:** physiological, regular rhythm without atrial contraction, and physiological, regular rhythm with atrial contraction; **B:** physiological, irregular rhythm without atrial contraction, and physiological, regular rhythm without atrial contraction; and **C**: physiological, irregular rhythm without atrial contraction and physiological, irregular rhythm with atrial contraction. Scatter points represent the results for each heart in the cohort. AF = atrial fibrillation; ctrl = control; LVEF = left ventricular ejection fraction; LVSV = left ventricular stroke volume; pharm. = pharmacologic; SR = sinus rhythm.

To investigate the improvement in LVEF achieved with different AF management strategies, we compared untreated AF simulations, representing preablation dynamics, with pharmacologic rate control (a physiological, irregular rate without atrial contraction), paced rate control (a physiological, regular rate without atrial contraction), and rhythm control (a physiological, regular rate with atrial contraction). Figures 3A and 3B and Table 3 present this comparison. It is assumed that paced rate control leads to physiological activation but that patients managed with pharmacologic rate control may continue to experience variance in R-R interval.

**Table 3:** Simulated LVSV and LVEF for AF and clinically relevant outcomes

Simulated rhythm	LVSV [mL]	LVEF [%]	LVSV vs AF [%]	D LVEF∗ vs AF [%]
Untreated AF	50.0 ± 12.2	48.6 ± 3.5	-	-
Paced rate control	53.1 ± 13.3	51.2 ± 3.1	+6.0 ± 1.1†	+2.6 ± 0.4†
Pharmacologic rate control	53.4 ± 13.1	51.4 ± 3.3	+6.8 ± 0.7†	+2.8 ± 0.3†
Rhythm control	61.6 ± 15.2	53.8 ± 3.5	+23.3 ± 2.9†	+5.1 ± 0.4†
Sinus rhythm with irregularity	53.2 ± 13.0	51.3 ± 3.3	+6.4 ± 0.7†	+2.7 ± 0.2†

AF = atrial fibrillation; LVEF = left ventricular ejection fraction; LVSV = left ventricular stroke volume.

∗ D LVEF is an absolute percentage change.

† Denotes significance with *P* < .01 after Bonferroni correction.

The average simulated values for LVEF increased from 48.6% ± 3.5% in untreated AF to 51.4% ± 3.3%, 51.2% ± 3.1%, and 53.8% ± 3.5%, for pharmacologic rate control, paced rate control, and rhythm control, respectively. LVSV increased by +6.8% ± 0.7%, +6.0% ± 1.2%, and 23.3% ± 3.0% for pharmacologic rate control, paced rate control, and rhythm control, respectively. No significant differences between sexes were found when comparing the improvement achieved with paced rate control, pharmacologic rate control, or rhythm control (vs untreated AF). Rhythm control had the greatest impact on LV function compared with untreated AF. We predict that paced and pharmacologic rate control have an equivalent impact on LV function given heart rate irregularity had no significant impact on LV function at a physiological heart rate.

### Interaction between atrial contraction and regular rhythm

The factorial study design also allowed the interaction between atrial contraction and regular rhythm to be explored. To quantify the contribution of effective atrial contraction in the presence of an irregular rhythm, we varied atrial contraction in simulations with irregular and physiological heart rates. Figure 3C compares the simulated LVEF for untreated AF, irregular rhythm without effective atrial contraction, and irregular rhythm with effective atrial contraction (average LVEF of 48.6% ± 3.5%, 51.4% ± 3.3%, and 51.3% ± 3.3%, respectively). Effective atrial contraction in the presence of irregular rhythm has an equivalent effect on LV function to that of pharmacologic or paced rate control. This indicates that the benefit derived from atrial contraction may be dependent on the degree of irregularity in the heart rate and may have implications for patients experiencing atrial ectopy after AF treatment.^40^

## Discussion

We quantified the independent and synergistic effects of heart rate, rhythm regularity, and effectiveness of atrial contraction on LV function in a cohort of 10 virtual patients with AF using computer simulations. Our results show that physiological ventricular rate, regular rhythm, and effective atrial contraction in isolation all contribute significantly to healthy LVEF and SV. However, the interaction among these factors is complex. Rate control significantly improves LV function compared with untreated AF, independently of heart rate regularity. Rhythm control also significantly improves LV function compared with untreated AF, although the benefit conferred by restoring atrial contraction may be dependent on the regularity of the rhythm.

### Rate, regularity, and atrial contraction all contribute to healthy LV function

The relationship between heart rate and SV is complex. Although some studies have found an increase in SV with increased heart rate,^41^ studies including patients with AF have seen a decrease in SV with increased heart rate.^42^ Consistent with experimental pacing studies, we found a 10.4% decrease in LVSV function when increasing heart rate by 30 bpm. Stojadinović and others^14^ observed an 11% decrease in LVSV when increasing the rate by 40 bpm. The volume transients produced by the simulations (Supplemental Material 11) show that the increased time for filling at slow rates allows the heart to reach a greater LV EDV (SR: 116.2 ± 32.6 mL vs increased rate: 113.4 ± 32.5 mL, *P* < .01). This efficiently preloads the ventricles, causing them to contract more effectively owing to the Frank-Starling mechanism^43^ (Supplemental Material 11) and reach a smaller LV end-systolic volume (SR: 54.6 ± 18.4 vs increased rate: 58.2 ± 19.8, *P* < .01). The combination of a decreased LV EDV and increased LV end-systolic volume at faster heart rates leads to a significant decrease in SV and EF.

Similarly, Stojadinović and others observed a decrease in LVSV of 10.1% when pacing from the atrioventricular node (thereby avoiding atrial contraction) at the same rate, compared with the 14.0% decrease observed in our simulations. This decrease is caused by the loss of the “atrial kick,” causing a decrease in the LV EDV (SR: 116.2 ± 32.6 mL vs increased rate: 105.1 ± 29.4 mL, *P* < .01) when atrial contraction is removed. As with the effect of increased rate, this decrease in LV EDV will limit the contribution of the Frank-Starling mechanism to LV ejection.

Finally, we found a decrease in LVSV of 13.7% with an irregular heart rate. This aligns with the 15.4% decrease (in cardiac output) observed with irregular right ventricular pacing^44^ that was hypothesized to be due to the Frank-Starling mechanism. All 3 AF mechanisms compromise LV function, and all affect LV function, at least in part, through the Frank-Starling mechanism.

### Rhythm control is superior to rate control

We find a significant improvement in LV function with rhythm control compared with rate control. This suggests a possible mechanism to explain the EAST-AFNET 4 trial,^8^ which found a lower risk of adverse cardiovascular outcomes with early rhythm control, which our results would explain through improved LV function. Similarly, our observed improvement of LV function with rhythm control is consistent with the CASTLE-AF trial,^9^ which found that in patients with advanced heart failure, rhythm control through catheter ablation was associated with a lower risk of death or worsening heart failure than was medical therapy (pharmacologic rate or pharmacologic rhythm control). This aligns with our findings that rhythm control is superior to rate control but suggests that this observation may be obscured in clinical trials using antiarrhythmic drugs, which are poorly tolerated and frequently unsuccessful.^3^^,^^41^

### Rhythm control may not benefit all patients equally

Our findings indicate that an improvement in LV function will not be achieved in all patients by restoring regular rhythm or atrial contraction. If medical rate control achieves a physiological but irregular rate, our simulations predict that there would be limited to no benefit to LV function in restoring a regular heart rate. If rhythm control achieves a physiological and regular heart rate but the atria cannot contract, the response will be comparable to rate control. Our results showed that rhythm control requires both atrial contraction and regular rhythm to improve LV function compared with rate control. This implies that there will be no benefit associated with restoring SR in cases when restoring atrial contraction is unlikely to be viable or there is a high risk of atrial ectopic beats.^40^ This may have implications for selecting appropriate therapies for patients with a large, dilated atria, high fibrosis burden in the atria,^45^ or significant ablation scarring,^46^ and warrants further research.

### Limitations

We used whole-heart electromechanics modeling to investigate the effect of different AF management strategies on LV function. Despite its significant contribution, there are some limitations to this research. Firstly, only 2 irregular rhythms were simulated—1 each sampled from AF ECGs with high and low ventricular rates. This means the effect of more or less irregular rhythms has not been investigated, although previous studies have not found significant differences.^14^ We did not specifically investigate the hemodynamic effect of ectopic beats and their locations. We also assumed that physiological ventricular activation could be achieved with AV node ablation and pacing and did not consider different pacing systems. Furthermore, this study only investigated the effects of AF at 2 heart rates and did not extend the factorial study across the whole cohort to include a very fast ventricular rate. However, further investigation in a single heart model suggests the effects are replicated at heart rates of 120 bpm (Supplemental Material 12). The investigation of rate control was limited to the hemodynamic effects of limiting rate; the possible negative inotropic effects of β blockers are explored in Supplemental Material 13. The model also does not account for the long-term effects of AF on mitral and tricuspid valve regurgitation due to atrial dilation.^47^ Finally, because patient-specific data for different levels of atrial contraction and/or ventricular rates were not available, the model could not be calibrated for each specific patient. However, the model was calibrated and validated against literature data, showing that the model replicates physiological behavior under different conditions.

## Conclusion

Physiological ventricular rate, regular rhythm, and effective atrial contraction all significantly contribute to LV function. Successful rhythm control achieves superior LV function improvement to that of rate control in patients with AF. However, the additional benefits conferred by rhythm control might be achievable only in patients in whom both effective atrial contraction and regular rate can be restored. In all other patients (ie, those with a high interstitial fibrosis from AF,^45^ fibrosis from excess ablation,^46^ or high levels of ectopic activity^40^), pharmacologic rate control might constitute a comparable AF management strategy.

## Disclosures

G.P., C.M.A., and E.J.V. are cofounders of NumeriCor GmbH. The remaining authors have no conflicts of interests to disclose.
