# Supplementary material for: Rhythm control benefits left ventricular function compared with rate control in patients with atrial fibrillation: A computational study
Source: Heart Rhythm O2. 2025 May 9;6(7):1011–9. doi: 10.1016/j.hroo.2025.04.014 (PMC12302171; doi:10.1016/j.hroo.2025.04.014)
Supplement: Supplementary Materials [file mmc1.docx]

**Supplementary materials**

**Abbreviations**

AF – atrial fibrillation

APD – action potential duration

BB – Bachmann bundle

BCL – basic cycle length

bpm – beats per minute

CaMKII - calcium/calmodulin-dependent protein kinase II

CV – conduction velocity

ECG - electrocardiography

EDV – end-diastolic volume

EF – ejection fraction

FEC – fast endocardial conduction

HF – heart failure

HM – history matching

GPE – Gaussian process emulator

GSA – global sensitivity analysis

LA – left atrium

LV – left ventricle

P-V – pressure-volume

RA – right atrium

RV – right ventricle

SERCA - sarco/endoplasmic reticulum ATPase

SV – stroke volume

SR – sinus rhythm

TAT – total activation time

**1: Calibration methods**

This study required calibration of multiple models: the calcium transient of the modified atrial cell model; the activation times of the atria and ventricles; and the hemodynamic behavior of the whole-heart model. The calibration for each of these models made use, in part or in full, of Gaussian process emulators (GPEs), global sensitivity analysis (GSA) and history matching (HM), based on the approach of Longobardi et al.^1^ These methods have been comprehensively explained and applied in previous four chamber heart model studies.^2^

**2: An update on the Courtemanche model to include the effect of CaMK-II on uptake to the sarcoplasmic reticulum in atrial myocytes**

The Courtemanche model^3^ has been used extensively in modelling of the atrial action potential.^4–6^ However, the Courtemanche model does not display physiological rate dependency therefore limiting its use in simulations. **Figure 1** shows the Ca^2+^ and tension transients produced by the Courtemanche model at pacing frequencies of 1 Hz, 1.5 Hz and 2 Hz. As the pacing frequency increases, there is a significant decrease in the peak intracellular Ca^2+^ and the peak tension. This contrasts with experimental findings related to rate-dependency.

Grand et al. investigated the effect of pacing frequency on action potential duration (APD) in human atrial myocytes and found a decrease in the APD with decreasing cycle length.^7^ Maier et al. investigated calcium handling in the isolated human atrial myocyte^8^ and studied the effect of pacing frequency on intracellular Ca^2+^ via the photoprotein aequorin. An increase in the pacing frequency from 1 Hz to 2 Hz caused an increase in the peak intracellular Ca^2+^ of approximately 30 %. An appropriate ionic model for human atrial action potential should, therefore, display both characteristics - a decrease in APD and an increase in peak intracellular Ca^2+^ with increasing pacing frequency.


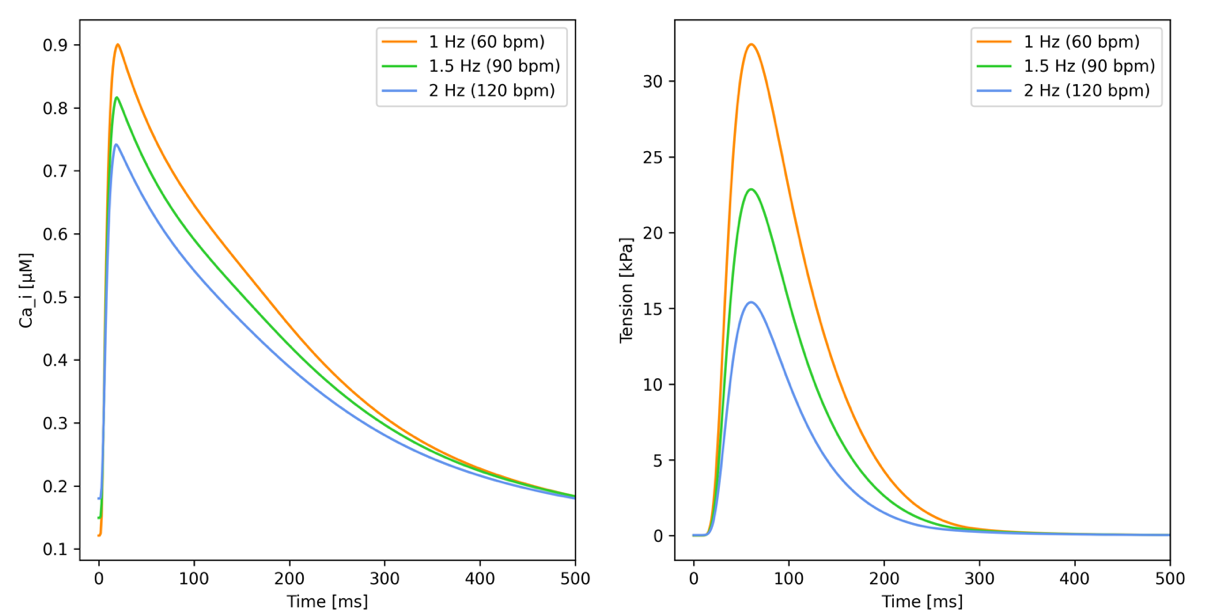


**Figure 1: Ca^2+^ and tension transients for the Courtemanche model at pacing frequencies of 1 Hz, 1.5 Hz and 2 Hz.** The peak of both transients decreases with increasing pacing frequency, in contrast to the expected physiological behavior.

We propose a modified version of the Courtemanche model which includes a Ca^2+^/calmodulin-dependent protein kinase II (CaMK-II)-mediated sarco/endoplasmic reticulum ATPase (SERCA) current to achieve sufficient re-uptake of Ca^2+^ to the sarcoplasmic reticulum at higher pacing frequencies. The formulations for the SERCA current are taken from the Gattoni model^9^ of the rat ventricular myocyte. The formulations for all other currents within the Courtemanche model were left unchanged. The modified model will be referred to here as the Courtemanche-CaMK model.

To modify the Courtemanche model, the I_up_ current representing the uptake current of intracellular calcium to the sarcoplasmic reticulum (SR) was replaced with a CaMK-II-regulated SERCA current ($I_{SERCA}$):

$$I_{SERCA}=g_{SERCA}.\frac{\left[ Ca^{2+} \right]_{i}}{K_{SERCA}+ \left[ Ca^{2+} \right]_{i}}$$

where $g_{SERCA}$and $K_{SERCA}$are the maximum pump rate and the half-saturation of the SERCA pump, respectively. $g_{SERCA}$was then regulated by the fractional occupancies of active CaMK-II ($f_{a}$), bound CaMK-II ($f_{b}$) and trapped CaMK-II ($f_{t}$):

$$g_{SERCA}=\bar{{}_{gSERCA}} .\frac{f_{a}}{K_{m, CaMK}-f_{a}}$$

$$f_{a}=f_{b}+f_{t}$$

$$\frac{df}{dt}= f_{b}\left( f_{b}+f_{t} \right)-f_{t}$$

$$f_{b}=f_{0}\left( 1-f_{t} \right)\frac{1}{1+ \frac{K_{m, CaM}}{\left[ Ca^{2+} \right]_{i}}}$$

where $\bar{{}_{gSERCA}}$ is the maximum increase in $g_{SERCA}$ due to CaMK-II, $K_{m, CaMK}$ is the half saturation coefficient, and are the phosphorylation and dephosphorylation rates of CaMK-II, respectively, and $f_{0}$is the fraction of active CaMK-II binding sites at equilibrium. $K_{m, CaM}$ is the Ca^2+^concentration at half-maximum CaMK-II activation.

$I_{SERCA}$ was integrated into the existing Courtemanche model via the derivative of the Ca^2+^ concentration in the uptake compartment ([Ca^2+^]_up_) and the derivative of the intracellular Ca^2+^ concentration:

$$\frac{d\left[ Ca^{2+} \right]_{up}}{dt}=I_{SERCA}-I_{up,leak}-I_{tr}.\frac{V_{rel}}{V_{up}}$$

$$\frac{d\left[ Ca^{2+} \right]_{i}}{dt}= \frac{B1}{B2}$$

$$B1=\frac{2I_{NaCa}-I_{p,Ca}-I_{Ca,L}-I_{b,Ca}}{2FV_{i}}+\frac{V_{up}\left( I_{up,leak}-I_{up} \right)+I_{rel}V_{rel}}{V_{i}}$$

$$B2=1+ \frac{\left[ Trpn \right]_{\max} K_{m,Trpn}}{\left( \left[ Ca^{2+} \right]_{i}+K_{m,Trpn} \right)^{2}}+ \frac{\left[ Cmdn \right]_{\max} K_{m,Cmdn}}{\left( \left[ Ca^{2+} \right]_{i}+K_{m,Cmdn} \right)^{2}}$$

for which the meanings and formulations of the currents are identical to those in the original Courtemanche model. The new parameters introduced into the model and their default values were as follows: = 0.05 ms^-2^, = 0.0002 ms^-1^, $f_{0}$, $\bar{{}_{gSERCA}}$ = 0.001158 mM/ms, $K_{m, CaM}$ = 0.0007 mM and $K_{m, CaMK}$ = 2.224. $K_{SERCA}$ (= 0.00092 mM) replaces $K_{up}$ (half-saturation constant for SR Ca^2+^ uptake) from the original Courtemanche model.

The Courtemanche-CaMK model was calibrated to achieve physiological Ca^2+^ dynamics at basic cycle lengths of 1000 ms (60 bpm) and 500 ms (120 bpm) using GPEs and HM. The following features of the calcium transient were considered in the calibration process:

1. Concentration of diastolic calcium ([Ca]_diast_)
2. Amplitude of calcium transient ([Ca]_ampl_)
3. Maximum rate of change of calcium ([dCa/dt]_max_)
4. Time to reach 90% of calcium transient decay (RT_90_)

The meaning and values of the calibrated model parameters are displayed in **Table 1**.

**Table 1: Calibrated values and definitions for the parameters in the new Courtemanche-CaMK model.**

| **Courtemanche-CaMK parameters** | | |
| --- | --- | --- |
| **Parameter** | **Value** | **Meaning** |
| g_Ca,L_ | 0.1401 | Max. conductance of the L-type Ca^2+^current |
| g_K1_ | 0.09 | Max. conductance of inward rectifier K^+^ current |
| g_Kr_ | 0.0294 | Max. conductance of rapid delayed rectifier K^+^ current |
| g_Ks_ | 0.129 | Max. conductance of slow delayed rectifier K^+^ current |
| g_Na_ | 7.8 | Max. conductance of fast inward Na^+^ current |
| g_b,Ca_ | 0.001519 | Max. conductance of background Ca^2+^ current |
| g_b,Na_ | 0.000674 | Max. conductance of background Na^+^ current |
| g_to_ | 0.1652 | Max. conductance of transient outward K^+^ current |
| k_rel_ | 30 | Max. Ca^2+^ release rate from the JSR |
| [Cmdn]_max_ | 0.05 | Total calmodulin concentration in myoplasm |
| [Csqn]_max_ | 10 | Total calsequestrin concentration in JSR |
| [Trpn]_max_ | 0.08102 | Total troponin concentration in myoplasm |
| I_NaCa,max_ | 2263 | Scaling factor for Na^+^/ Ca^2+^ exchanger current |
| I_NaK,max_ | 0.6 | Max. Na^+^/ K^+^-pump current |
| I_pCa,max_ | 0.275 | Max. sarcoplasmic Ca^2+^ pump current |
| I_up,max_ | 0.00572 | Max. uptake rate for Ca^2+^ into the NSR |
| τ_tr_ | 180 | Ca^2+^ transfer time constant |
| [Ca^2+^]_o_ | 1.8 | Extracellular concentration of Ca^2+^ |
| [K^+^]_o_ | 5.4 | Extracellular concentration of K^+^ |
| [Na^+^]_o_ | 140 | Extracellular concentration of Na^+^ |
| A_ch_ | 10^-6^ | Acetylcholine-activated K currents |
| α | 0.02948 | phosphorylation rate of CaMK-II |
| β | 0.0002793 | dephosphorylation rate of CaMK-II |
| f_0_ | 0.0542589 | fraction of active CaMK-II binding sites at equilibrium |
| ${}_{\mathrm{gSERCA}}$ | 0.012605 | maximum increase in $g_{SERCA}$ due to CaMK-II |
| $K_{m, CaMK}$ | 1.12575 | half saturation coefficient for CaMK-II |

The rate-dependency of the Courtemanche-CaMK model is shown in **Figure 2.** An increase in the peak intracellular Ca^2+^ and peak tension is seen with increasing pacing frequency. The model therefore successfully produces the rate-dependency observed in literature and is appropriate for use in simulations with varying heart rates.

**
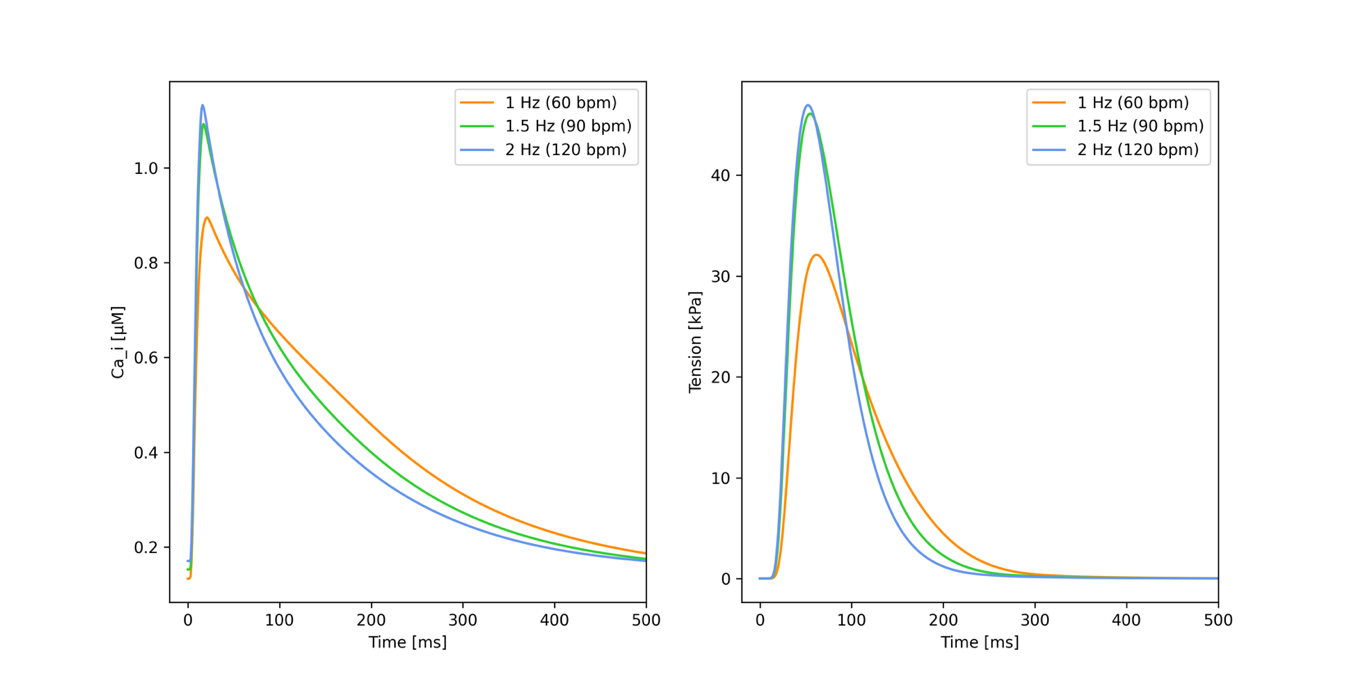
**

**Figure 2: Ca^2+^ and tension transients for the Courtemanche-CaMK model at pacing frequencies of 1 Hz, 1.5 Hz and 2 Hz.** The peak of the Ca^2+^ transient now increases with increasing pacing frequency, in line with experimental observations.

**3: Electrophysiology calibration**

Four parameters for tissue conduction velocities (CV) were calibrated to achieve physiological activation times for AF patients (ventricles: 102 ± 22 ms; atria: 112 ± 24 ms)^10,11^: CV in the fiber direction for the atria and ventricles (CV_f,v_ and CV_f,a_, respectively), a parameter scaling CV in the FEC layer based on CV_f,v_ (k_FEC_), and a parameter scaling CV in the Bachmann’s bundle based on CV_f,a_ (k_BB_). The parameter space was sampled with a Latin hyper cube design based on the ranges given in **Table 2**.

The reaction-eikonal model was used to simulate activation and the total activation times in the atria and ventricles were calculated (TAT_a_ and TAT_v_, respectively). These outputs were then used to train GPEs to predict TAT_a_ and TAT_v._ for a dense sampling in the parameter space (N = 10^5^). The optimal parameter set was selected by minimizing the sum of the normalized squared error between the target and expected values of TAT_a_ and TAT_v_.

All hearts in the cohort were calibrated to achieve the target values of TAT_a_ and TAT_v_. The fitted parameter values across the cohort were: CV_f,v_ = 0.66 ± 0.05 m/s, k_FEC_ = 4.22 ± 2.14, CV_f,a_ = 0.91 ± 0.08 m/s, k_BB_ = 3.44 ± 1.14. Electrical activation for one heart in the cohort is shown in **Figure 3**.

**Table 2: Parameter ranges used for electrophysiology calibration of the cohort of heart models.**

| Tissue electrophysiology parameters | | |
| --- | --- | --- |
| Parameter | **Range** | **Meaning** |
| *CV_f,v_* | 0.38 – 0.8 m/s | Conduction velocity in fiber direction for ventricles |
| *k_FEC_* | 1.2 – 8.4 | Scaling factor for the CV of the fast endocardial conduction layer |
| *CV_f,a_* | 0.3 – 1.03 m/s | Conduction velocity in fiber direction for atria |
| *k_BB_* | 1 – 5.7 | Scaling factor for the CV of the Bachmann bundle |

**
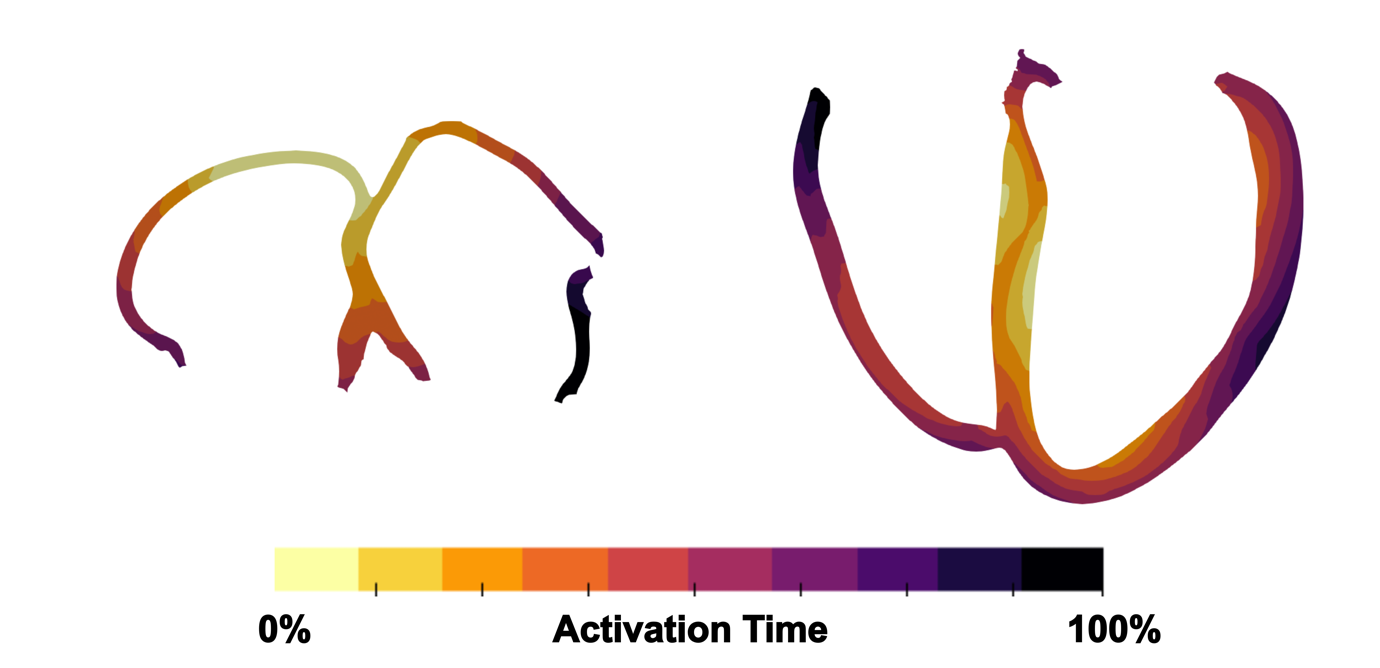
**

**Figure 3: Activation maps for the atria and ventricles, normalized by TAT_a_ and TAT_v_.**

**4: Electromechanical framework**

**
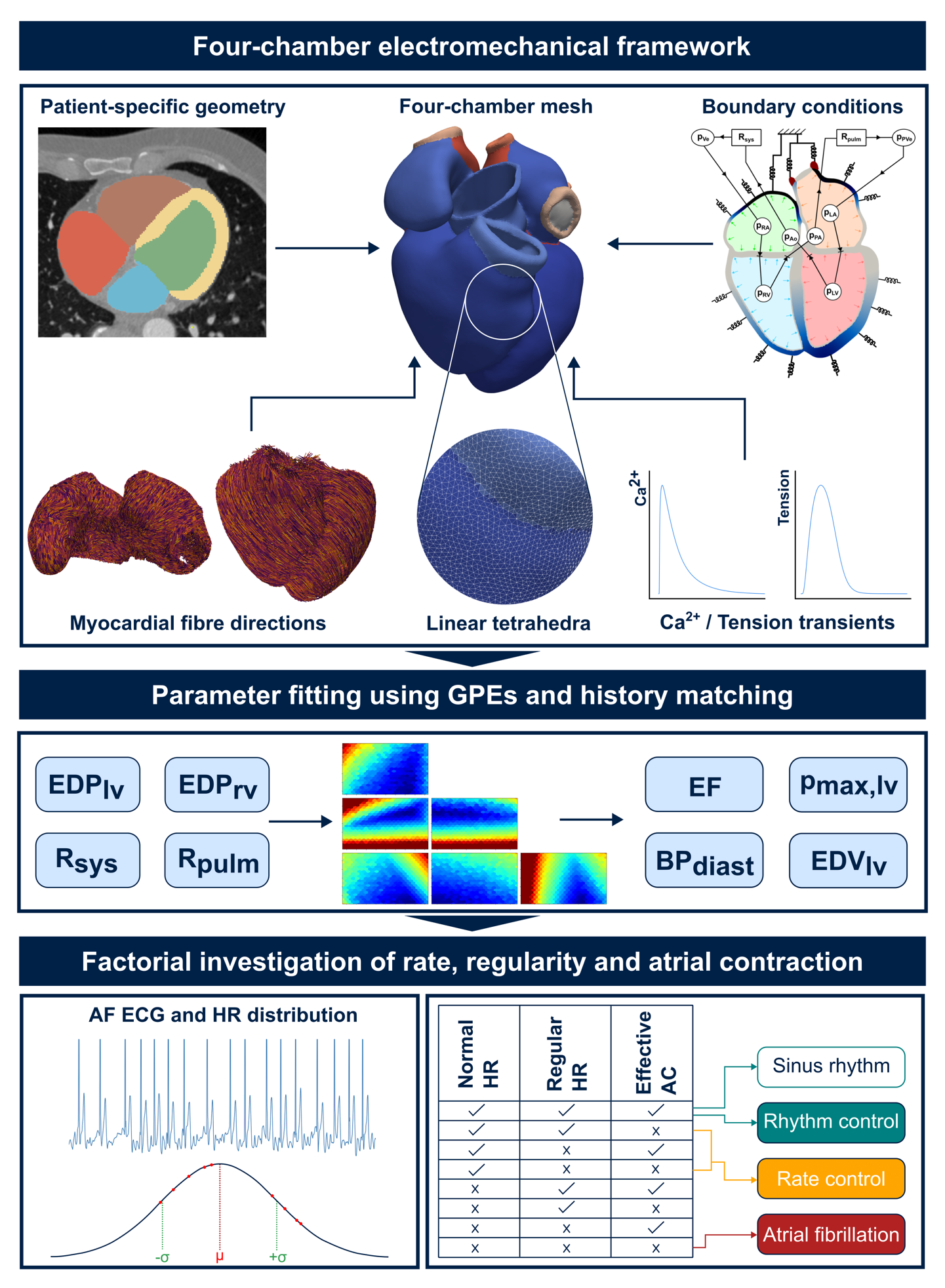
**

**Figure 4: Overview of the electromechanical framework used in four-chamber heart modeling**

**Patient-specific anatomies**

The four-chamber heart anatomies used in this study were generated from ECG-gated computed tomography of patients with AF. Whole heart images (in-plane resolution: 0.35 ± 0.02 mm, slice thickness: 0.6 mm) were acquired throughout the cardiac cycle and a segmentation of the end-diastolic image was used to generate a mesh with linear tetrahedra, using a previously published pipeline.^12,13^

**Atrial and ventricular fibers**

Ventricular myofiber orientations were assigned to each mesh using the Laplace-Dirichlet rule-based method by Bayer et al.^14^ The fiber direction varied from -60° to + 60° and the sheet direction varied from -65° to + 25° on the endocardium and epicardium, respectively.^14,15^ Atrial myofiber orientations were assigned to the endocardium and epicardium by mapping the Labarthe fiber atlas^16^ via universal atrial coordinates.^17^ An epicardial and endocardial layer of fibers were then assigned based on a transmural Laplace solution.

**Electrophysiology**

A reaction-eikonal model^18^ was used to simulate the electrical activation of the heart, leading to a solution for activation time $t_{a}(x)$ as a function of node location:

$$\sqrt{\nabla t_{a}\left( x \right)^{T}V\left( x \right)\nabla t_{a}\left( x \right)}=1 x\in\Omega$$

$$t_{a}\left( x \right)=t_{0} x \in\Gamma$$

Atrial activation was initiated at a point behind the superior vena cava, to approximate the sinoatrial node. The atrioventricular delay was 100 ms for simulations during calibration (with a heart rate of 60 bpm).^19^ Ventricular activation was initiated at 5 points of early activation^20^, where the His-Purkinje system emerges into the ventricular endocardium.

Atrial and ventricular myocardium were simulated as transversely isotropic conduction media, with preferred conduction direction aligned with local myofiber orientation. To simulate physiological activation, two additional regions were defined: the Bachmann’s bundle in the atria and a fast endocardial conduction (FEC) layer in the ventricular endocardium.

**Cell dynamics**

Ventricular and atrial action potentials were simulated using the ToR-ORd and Courtemanche model, respectively^21,22^. The Courtemanche model was updated to include the effect of CaMK-II on uptake to the sarcoplasmic reticulum in atrial myocytes, as the original model is limited in its ability to correctly reproduce rate dependency (see **Section 3**). The intracellular calcium transients simulated by the models were then coupled with the ventricular and atrial variants of the Land model^23^ to compute an active tension transient, accounting for length and velocity dependence of contraction.

**Passive material properties**

Atrial and ventricular myocardium were modelled as transversely isotropic, hyperelastic and nearly incompressible material with the Guccione law.^24^ All other structures (aortic wall, pulmonary artery and valve planes) were modelled as Neo-Hookean materials, with parameters assigned based on previous studies.^2,25^ Incompressibility was enforced for all tissue types using a penalty method.^26,27^ Passive material parameters were fixed for all models in the cohort and are provided in **Table 3**.

**Table 3: Passive material parameters for the four-chamber models.**

| Passive material parameters | | |
| --- | --- | --- |
| Parameter | **Value** | **Meaning** |
| a_LV_ | 1.7 kPa | Bulk stiffness (LV) |
| a_RV_ | 2.55 kPa | Bulk stiffness (RV) |
| a_AA_ | 2.5 kPa | Bulk stiffness (atria) |
| b_f_ | 8.0 | Stiffness in fiber direction |
| b_fs_ | 4.0 | Stiffness in fiber-transverse shear planes |
| b_ft_ | 3.0 | Stiffness in transverse plane |
| k | 1000 kPa | Bulk modulus for incompressible tissue |
| c_vp_ | 1000 kPa | Neo-hookean material parameter (valve planes) |
| c_Ao_ | 26.6 kPa | Neo-hookean material parameter (aorta) |
| c_Pa_ | 3.7 kPa | Neo-hookean material parameter (pulmonary artery) |
| c_vr_ | 7.45 kPa | Neo-hookean material parameter (vein rings) |

**Boundary conditions**

Normal springs scaled in space simulated the effect of the pericardium, as validated in previous studies.^25^ Finally, the mechanical contraction was coupled with a closed-loop model for the circulatory system based on CircAdapt.^28,29^ Accounting for the mechanical effect of the pericardium and the coupling with the circulatory system allows the model to fully capture the mechanisms of atrioventricular interaction.

**Software**

All simulations were run with the Cardiac Arrhythmia Research Package (CARP)^30,31^ on a supercomputer on 512 cores.

**5: Regulatory Feedback**

There are several regulatory feedback mechanisms by which the cardiovascular system attempts to maintain blood pressure and cardiac output as heart rate changes.

One such feedback loop is the Frank-Starling mechanism, where increased venous return (preload) leads to greater myocardial stretch and stronger contraction. This mechanism helps maintain cardiac output and blood pressure and is included in the tension development model (the Land model). This effect is investigated further in Supplement 11.

Another important feedback loop is the baroreceptor reflex, which regulates blood pressure by:

- Increasing heart rate and vasoconstriction when blood pressure is low.
- Reducing heart rate and promoting vasodilation when blood pressure is high.

However, in AF, baroreflex function is impaired due to:

- Irregular heart rate disrupting normal feedback mechanisms.^32^
- Chaotic atrial activity overriding parasympathetic responses.
- Chronic AF leading to baroreceptor desensitization, reducing its ability to stabilize blood pressure.^33^

Therefore, it remains unclear how baroreflex function interacts with AF therapies, particularly rate-controlling treatments that limit the ability of the heart to adjust its rate dynamically. Additionally, evidence suggests that AF itself may impair baroreflex sensitivity over time.^33^

To explore these interactions, additional simulations have been conducted examining the potential impact of baroreflex modulation. Specifically, the effect of vasoconstriction and vasodilation (as the heart rate decreases with therapy) has been considered by decreasing and increasing the surface area of the systemic vessels in the model, respectively. However, the best approach to modeling the baroreflex in this context is unclear due to: interactions between AF therapies and the baroreflex function; possible long-term impairment of baroreflex sensitivity; and the possibility that these effects may be most pronounced in acute post-treatment phases, rather than chronically.

The results are shown in **Figure 5** and the LVEF and LV SV are summarized in **Table 4**. The effect of the baroreceptor reflex on the simulations is primarily seen in the peak left ventricular pressure. Any effect on the LVEF is minimal and is therefore unlikely to affect the conclusions drawn.

**Table 4: Summary of the simulated LVEF and LV SV when effects of the baroreceptor reflex are included.**

|  | **LV EF [%]** | **LV SV [mL]** |
| --- | --- | --- |
| **Rhythm control** |  |  |
| Baseline | 51.1 | 87.7 |
| 10% Vasoconstriction | 50.8 | 87.4 |
| 20% Vasoconstriction | 50.4 | 86.9 |
| 10% Vasodilation | 51.3 | 88.0 |
| 20% Vasodilation | 51.5 | 88.2 |
|  |  |  |
| **Paced rate control** |  |  |
| Baseline | 48.5 | 73.7 |
| 10% Vasoconstriction | 48.3 | 73.4 |
| 20% Vasoconstriction | 48.0 | 73.1 |
| 10% Vasodilation | 48.7 | 73.9 |
| 20% Vasodilation | 48.8 | 74.0 |
|  |  |  |
| **Table cont.** |  |  |
|  | **LV EF [%]** | **LV SV [mL]** |
| **Pharmacological rate control** |  |  |
| Baseline | 48.4 | 73.5 |
| 10% Vasoconstriction | 48.2 | 73.2 |
| 20% Vasoconstriction | 47.8 | 72.8 |
| 10% Vasodilation | 48.6 | 73.8 |
| 20% Vasodilation | 48.8 | 74.0 |


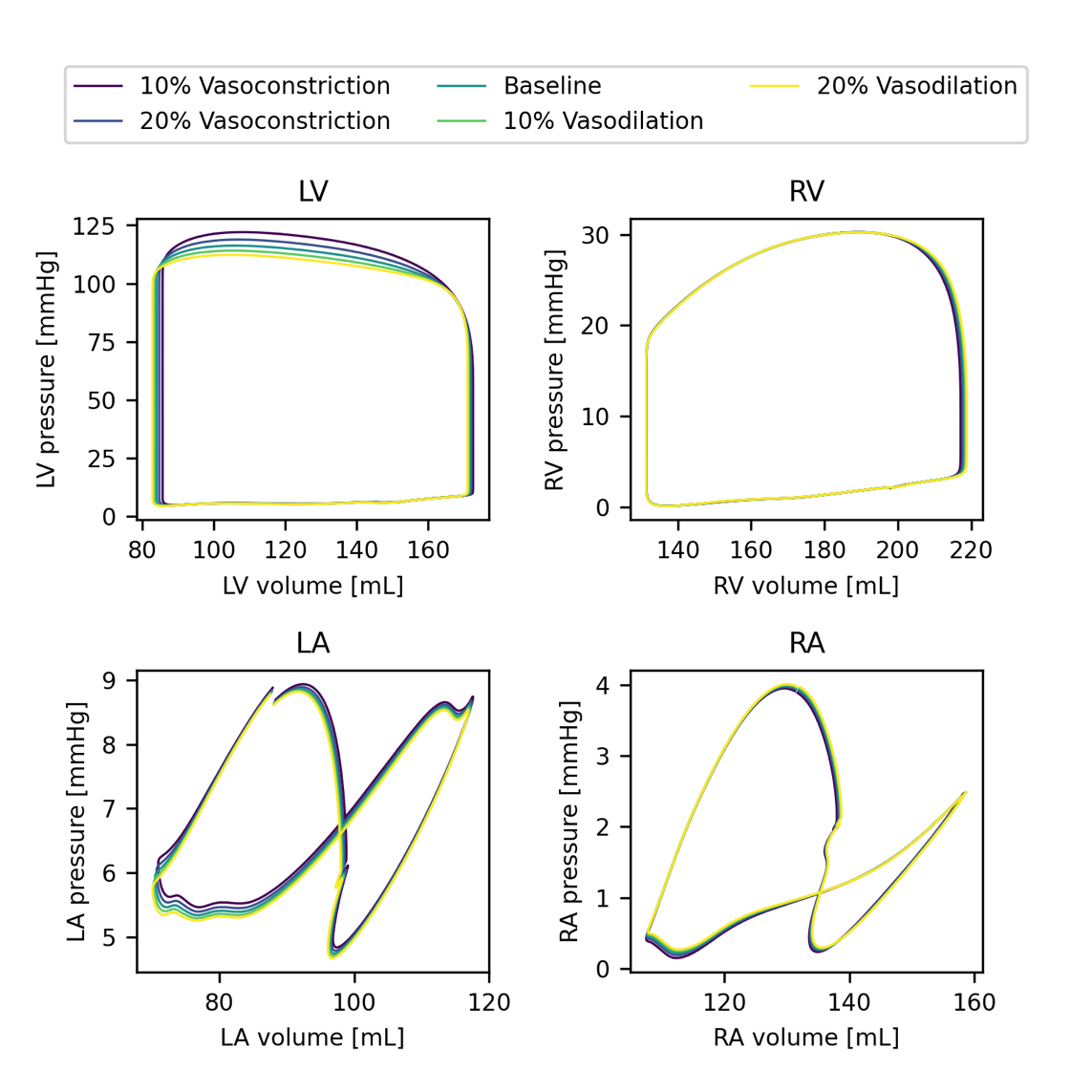


**Figure 5: Pressure-volume loops showing the effect of vasoconstriction and vasodilation in the system vessels during rhythm control, as an example.** Vasoconstriction/vasodilation has been modified in this way to model a potential contribution of the baroreceptor reflex to the results.

**6: Mechanics calibration**

The four-chamber models were calibrated to simulate physiological behavior and outputs during sinus rhythm. The criteria for calibration were defined as:

1. Systolic pressure in range 90-140 mmHg^34^
2. Diastolic pressure in range 60-90 mmHg^35^
3. Left ventricular ejection fraction (LV EF) > 50%^36^
4. Return to image-derived LV end-diastolic volume (LV EDV) ± 10%

In addition, calibrated models were checked to ensure correct morphology in the pressure-volume (P-V) loops of all four chambers and qualitatively correct motion of the heart with physiological atrioventricular plane displacement. GPEs and history matching were used to complete the calibration (see **Section 2**).

**Figure 6** shows the P-V loops in the four chambers for each heart model in the cohort. The expected morphologies of the P-V loops are seen in each model.


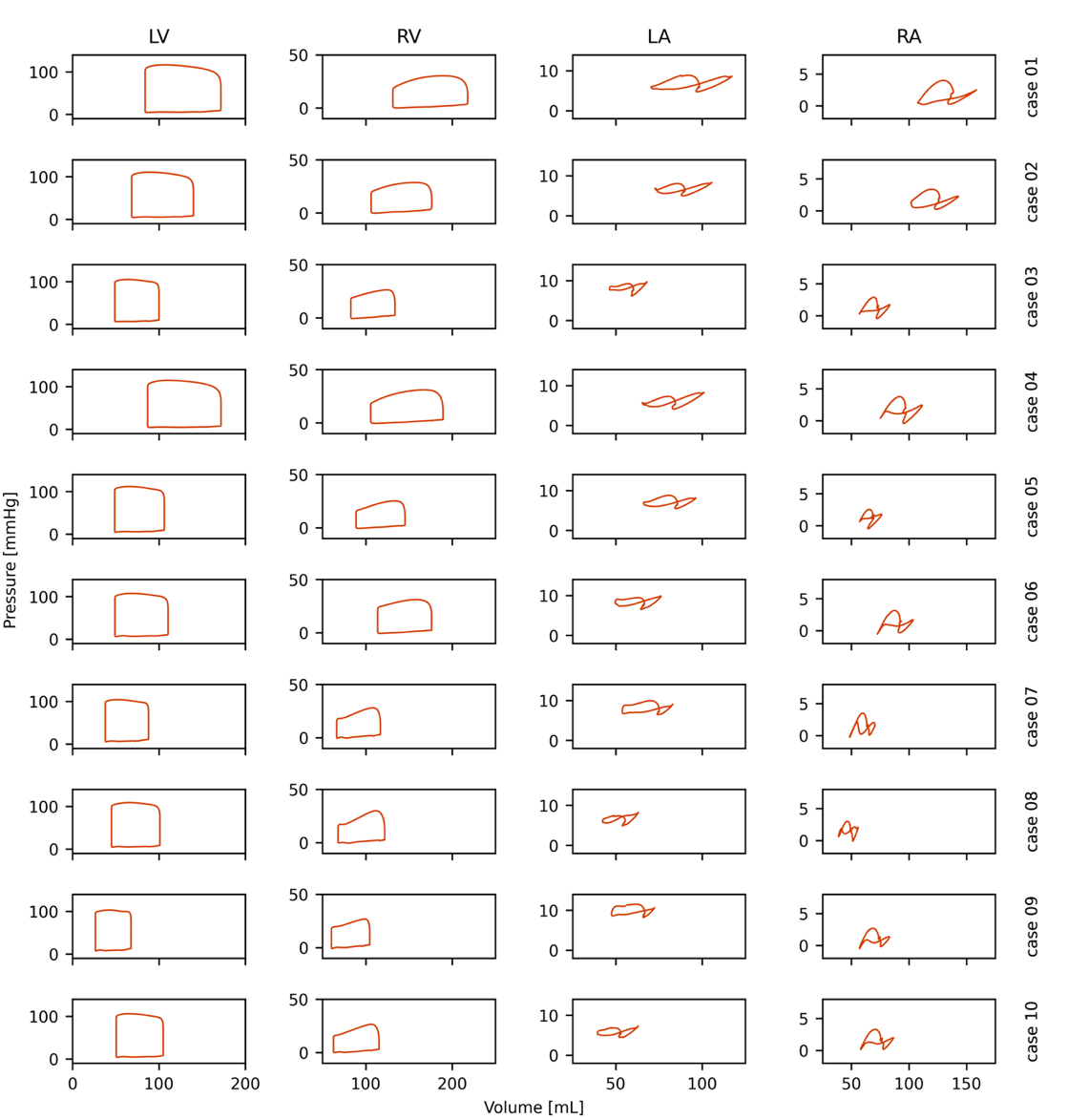


**Figure 6: P-V loops for the LV, RV, LA and RA for each of the 10 hearts in the cohort.** All heart models displayed the expected P-V morphology in each chamber, including the figure of eight in the atria showing atrial systole and venous return.

**7: R-R distributions for AF simulations**

An irregular rhythm is a characteristic feature of AF. To generate a set of basic cycle lengths (BCLs) for simulations with an irregular rhythm, two ECGs from AF patients with fast and slow mean heart rates were used to generate normal distributions.^37^ The distributions were then sampled to provide 9 values for BCL for the irregular simulations. Both ECGs were acquired over a 30 second period. **Figure 7A** shows a sample of the ECG with slow mean rate. **Figure 7B** shows the distribution of R-R intervals for the slow mean rate and the sampled values used as the BCLs in the slow irregular simulations (910, 790, 840, 1140, 890, 1190, 1180, 750, 1100 ms). **Figure 7C** shows the distribution of R-R intervals for the fast mean rate and the sampled values used as the BCLs in the fast irregular simulations (670, 600, 700, 660, 600, 630, 580, 710, 600 ms). The degree of irregularity can be summarized using the normalized standard deviation of R-R intervals (SDRR). The sampled BCLs have a mean of 977 ± 175 ms (SDRR = 17.9%) and 639 ± 48 ms (SDRR = 7.5%), respectively.

**
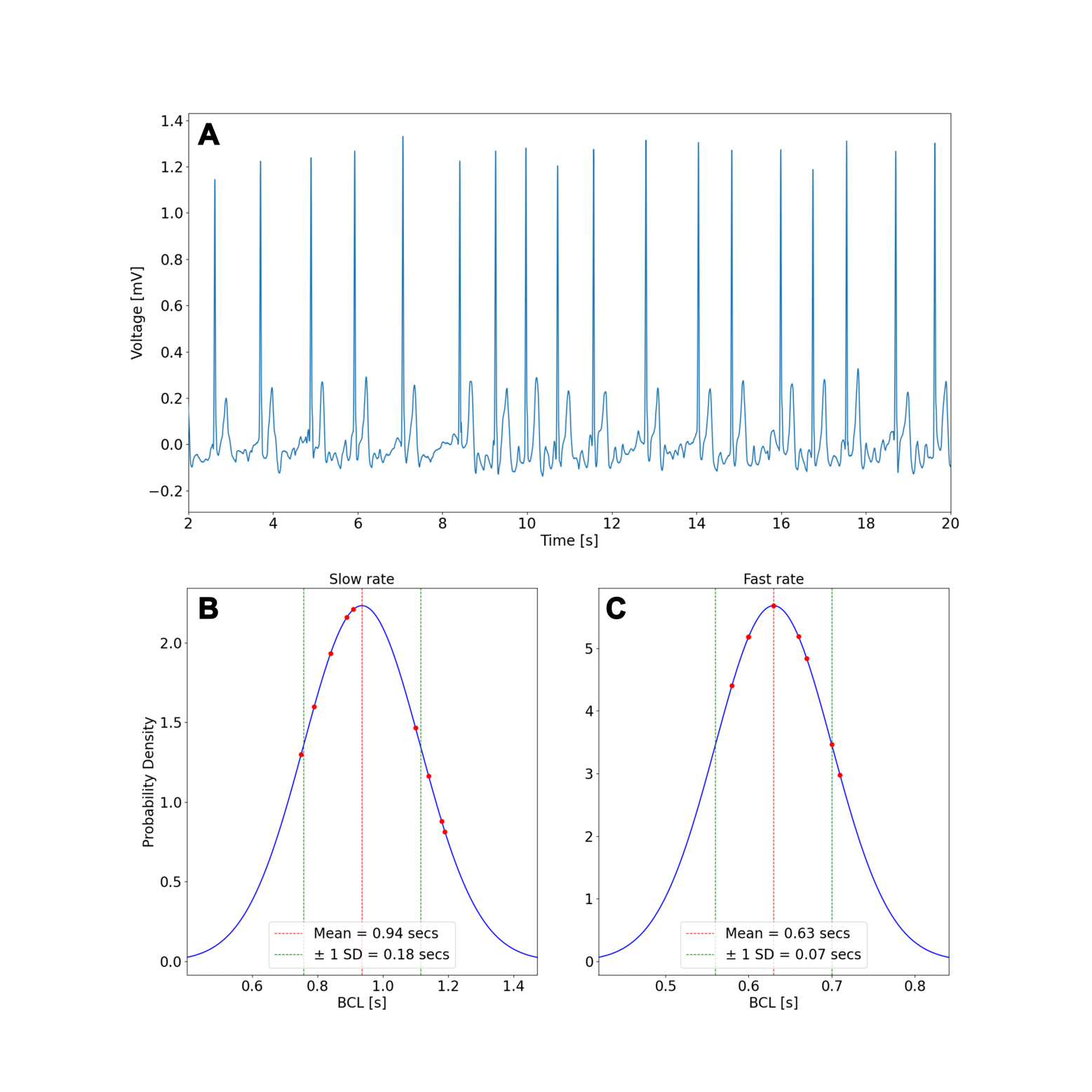
**

**Figure 7:** A) Example section from 30 sec AF ECG used to generate distribution of R-R intervals. B/C) Normally distributed R-R intervals generated from AF ECGs. Red points show the sampled R-R values.

**8: Factorial study – full results**

The clinically relevant results from the factorial study of heart rate, rate regularity and effective atrial contraction were presented in the main body of the work. In **Figure 8**, the full results of the factorial study are presented.

**
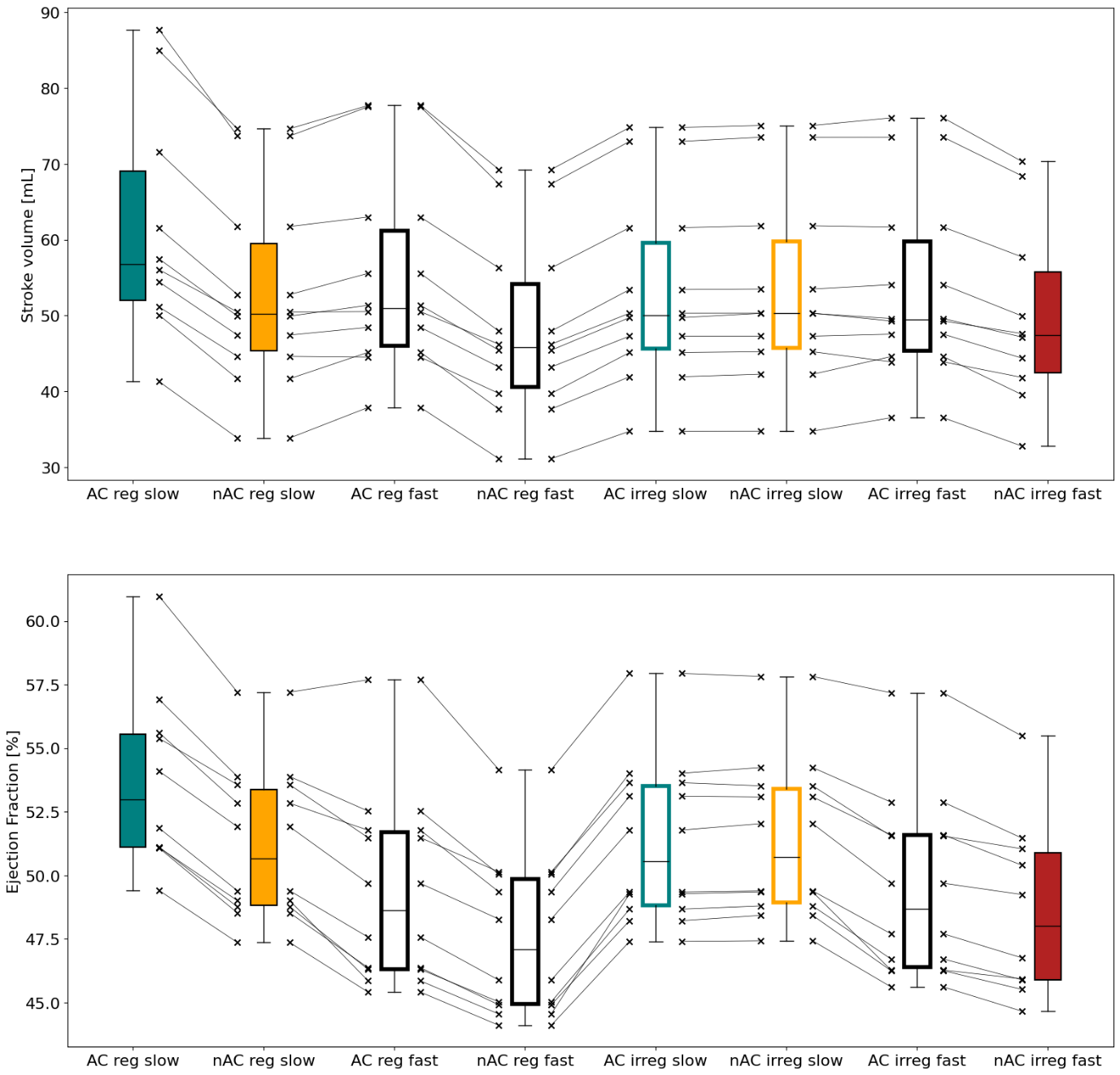
**

**Figure 8: Full results of the factorial study varying heart rate, rate regularity and atrial contraction.** Stoke volume (SV) and ejection fraction (EF) for each simulation are shown. Key: n/AC = no/ atrial contraction; reg/irreg = regular/irregular rate; fast/slow = fast/slow rate.

**9: Further Results – Pressures and Cardiac Output**

There are several metrics by which the performance of the heart can be analyzed. LVEF and LV SV are widely recognized as strong indicators of systolic function and heart failure, motivating their use in this paper to discuss the effects of anti-arrhythmic therapy in the context of progression to heart failure.^38^ However, other metrics are of interest and can be used to provide additional insights into the results of this study. For irregular rhythm simulations, the metrics here are the systolic/diastolic pressures (**Figure 9**) and cardiac output (**Figure 10**) averaged over the final 9 heart beats.

Cardiac output is the volume of blood pumped by the heart per minute. AF can be categorised either as “controlled” or “uncontrolled” depending on the change in the relation between cardiac output and ventricular rate. At ventricular rates slower than 90 bpm,^39^ AF is generally controlled and the cardiac output increases as the ventricular rate increases. At rates over 140 bpm, AF is generally uncontrolled and the cardiac output decreases as the ventricular rate increases. In the range 90-140 bpm, AF can be either controlled or uncontrolled. The rates used in these simulations (64 and 91 bpm) lie in the controlled AF range. The results (**Figure 10**) show that the simulations result in the correct relation between cardiac output and ventricular rate for intermediate heart rates.


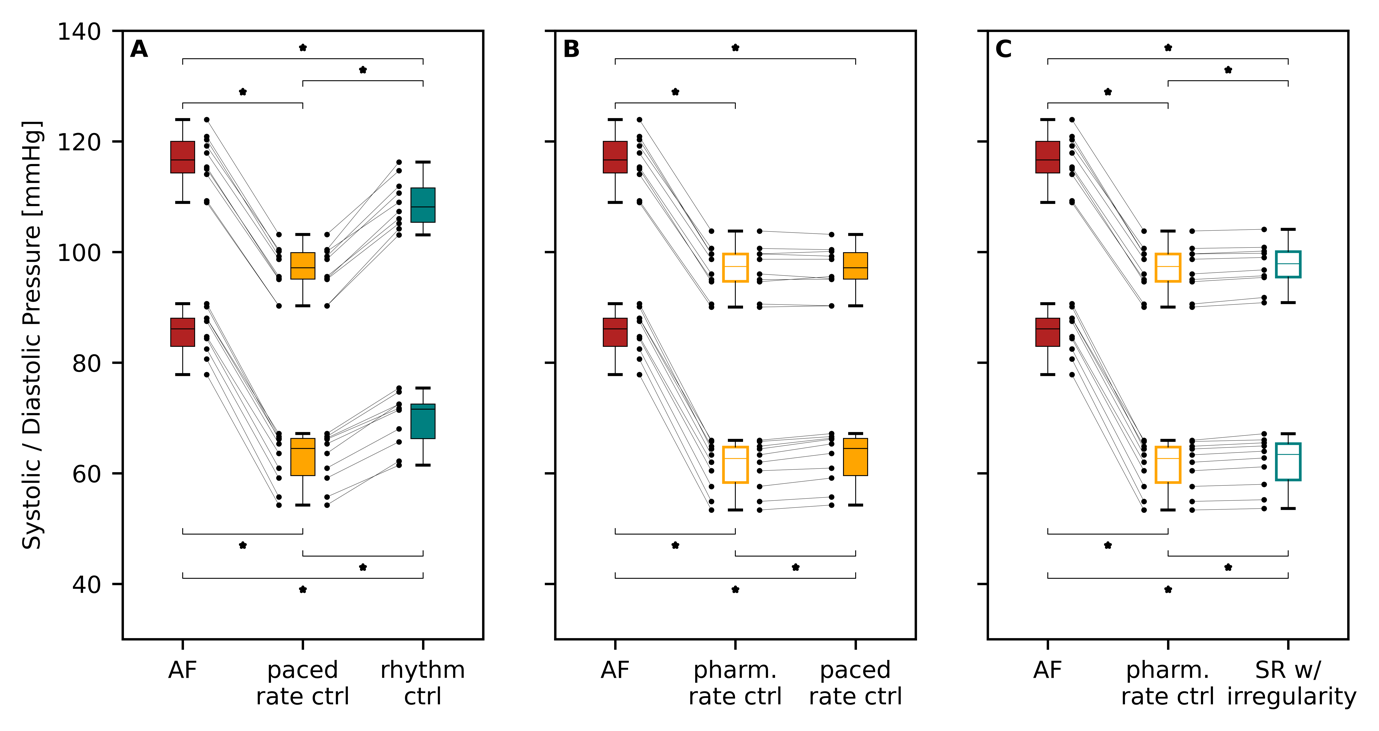


**Figure 9: Systolic and diastolic pressures.** The increased pressures simulated during AF align with observations of higher blood pressure at higher heart rates. **A**: Comparison of average systolic/diastolic pressures during AF, paced rate control and rhythm control. **B**: Comparison of average systolic/diastolic pressures during AF, pharmacological rate control and paced rate control. **C**: Comparison of average systolic/diastolic pressures during AF, pharmacological rate control and sinus rhythm with irregularity.


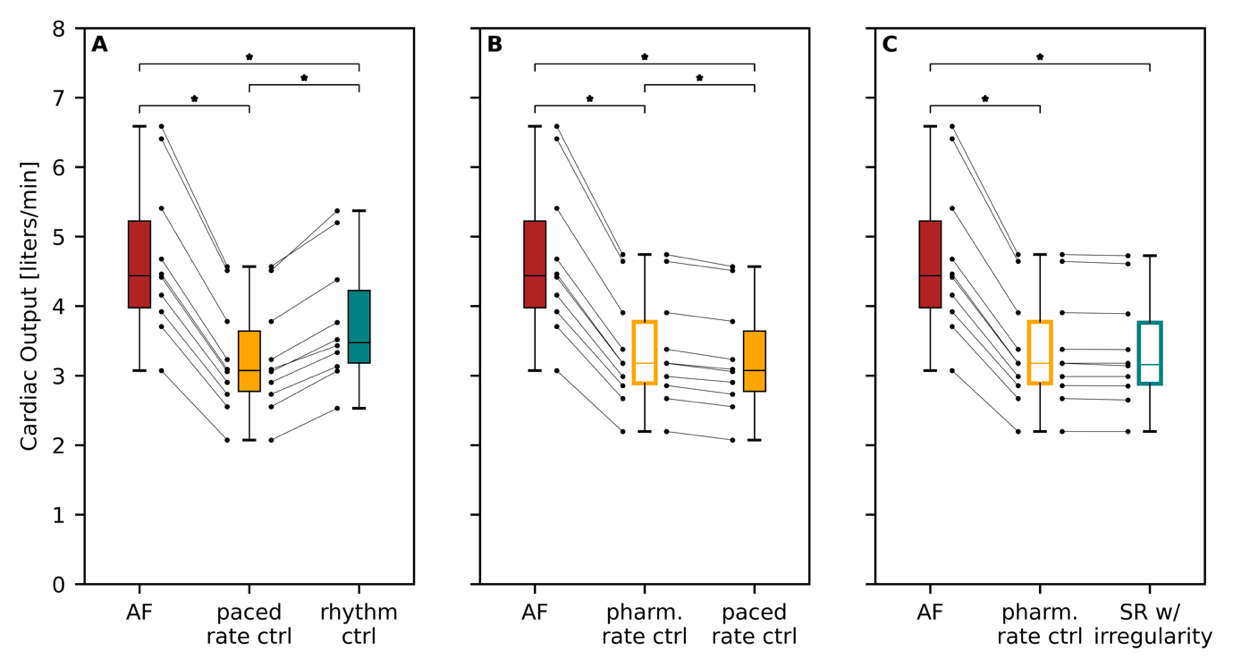


**Figure 10: Cardiac output.** Cardiac output is calculated as the LV SV multiplied by the heart rate in bpm. The increase in cardiac output with increasing heart rate in AF patients aligns with literature data for the range of heart rates being considered. **A**: Comparison of cardiac output during AF, paced rate control and rhythm control. **B**: Comparison of cardiac output during AF, pharmacological rate control and paced rate control. **C**: Comparison of cardiac output during AF, pharmacological rate control and sinus rhythm with irregularity.

**10: Sex differences in LV output**

The LVEF and LVSV results for AF and AF management simulations are reproduced in

**Figure 11**, with patient sex indicated on the figure. The female hearts (n=4) have a 29.5% smaller LV SV on average in SR than the male hearts (n=6), in line with the 23% smaller LVSV reported in the literature.^40^ No sex-specific values for cell or tissue parameters were included in this study so differences in LV output between sexes should be understood as only related to the geometry of the patient-specific meshes. No significant differences between sexes were found when comparing the improvement from AF achieved with paced rate control, pharmacological rate control, rhythm control or rhythm control with ectopy (compared using t-test with significance level of 0.05).


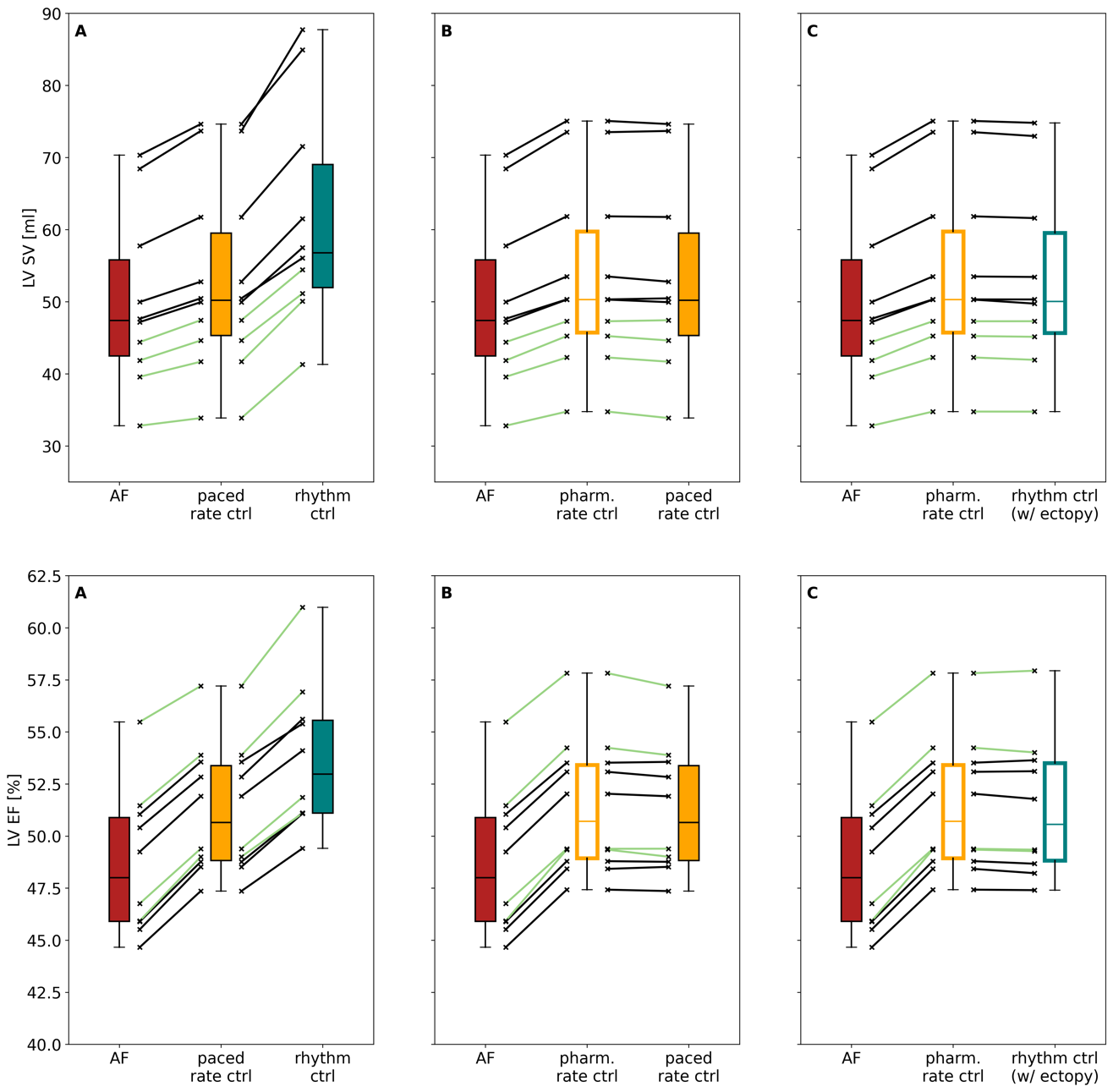


**Figure 11: The effect of different AF management strategies on LV function:** box plots of the LV SV and LV EF for simulated AF compared to simulations with: (A) slow, regular rhythm without atrial contraction and slow, regular rhythm with atrial contraction; (B) slow, irregular rhythm without atrial contraction and slow, regular rhythm without atrial contraction; (C) slow, irregular rhythm without atrial contraction and slow, irregular rhythm with atrial contraction. Scatter points represent the results for each heart in the cohort. Female and male sex are indicated with green and black lines, respectively.

**11: Contribution of Frank-Starling mechanism**

The increased time for filling at slow rates allows the heart to reach a greater LV EDV than at faster rates, as shown in **Figure 12**. It was hypothesized that this efficiently preloads the ventricles, causing them to contract more efficiently due to the Frank-Starling mechanism.^41^ This suggests that a smaller improvement in LV SV (SR vs AF) should be observed if length dependence is turned off in the model. We tested this hypothesis by setting the length dependence parameter in the Land model (*β_0_*) to zero for both the ventricular and atrial myocardium. Simulations for AF and SR were then repeated for one case and results for LV SV are shown in

**Table 5**. In the absence of the Frank-Starling mechanism, the percentage increase in the LV SV between AF and SR is smaller, indicating that the hypothesized effect of the Frank-Starling mechanism is correct.


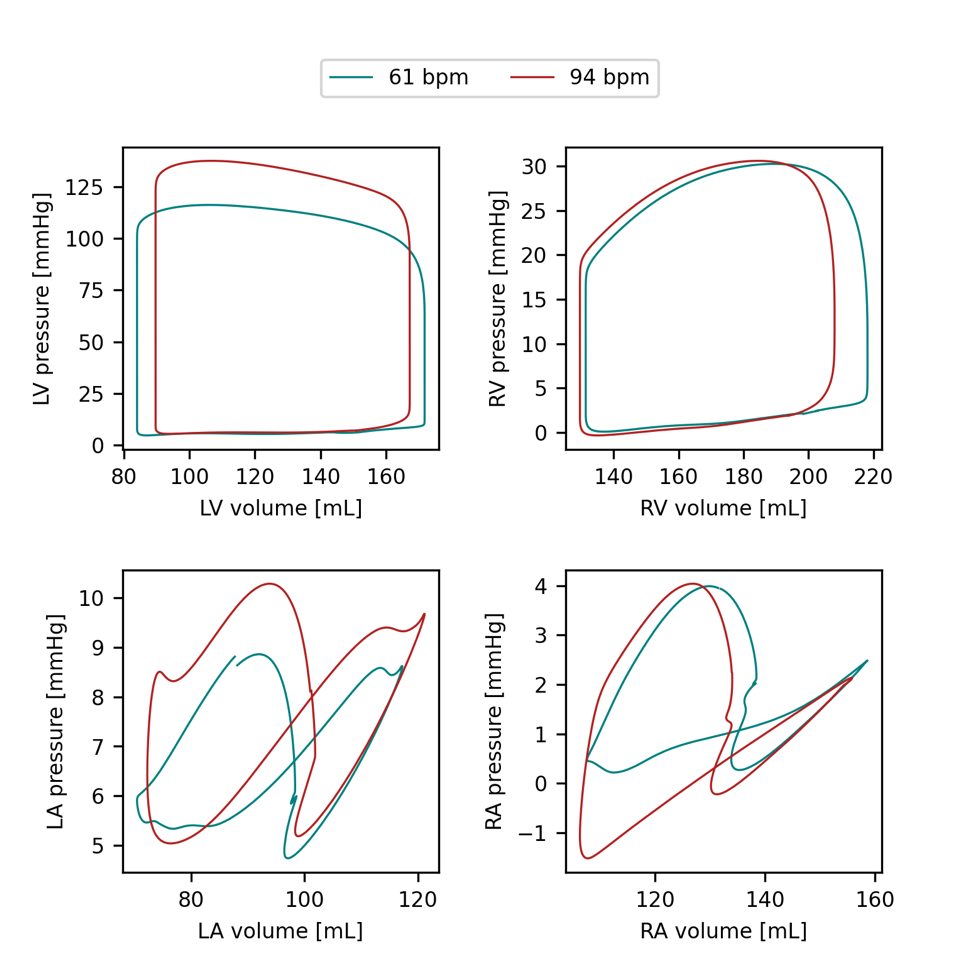


**Figure 12: Example pressure-volume loops for simulations with a slow and fast heart rate.** At a slower heart rate, the LV achieves a greater EDV and smaller ESV.

**Table 5: Simulated LV SV for AF and SR simulations with and without including the length-dependence effect of the Frank-Starling mechanism.**

|  | **LV SV [mL]** | | **% increase in LV SV (AF vs SR)** |
| --- | --- | --- | --- |
|  | **AF** | **SR** |  |
| **w/ Frank-Starling**  **(*β_0_* = 2.3)** | 68.4 | 87.7 | +28.2 % |
| **w/o Frank-Starling**  **(*β_0_* = 0)** | 78.1 | 98.3 | +25.8 % |

**12: Simulating a faster heart rate**

The two average heart rates (61 and 94 bpm) used in this study were calculated from AF patient ECGs. However, the average heart rate of patients with AF can be significantly faster. To investigate whether our results could be generalized to faster heart rates, we repeated the simulations with an average heart rate of 120 bpm on one heart model (case 07). The BCLs used in these simulations were 500, 470, 550, 520, 470, 550, 520, 470, 490, 450, 550, 470 and 500 ms. **Figure 13** shows the results of these simulations alongside previous simulations. Increasing the heart rate further acts to reduce the LV SV and LV EF. This suggests that AF patients with a faster average heart rate would experience greater benefit from rate or rhythm control. However, we did not see any significant interactions between irregularity and rate at 120 bpm that were not observed at 94 bpm.

**
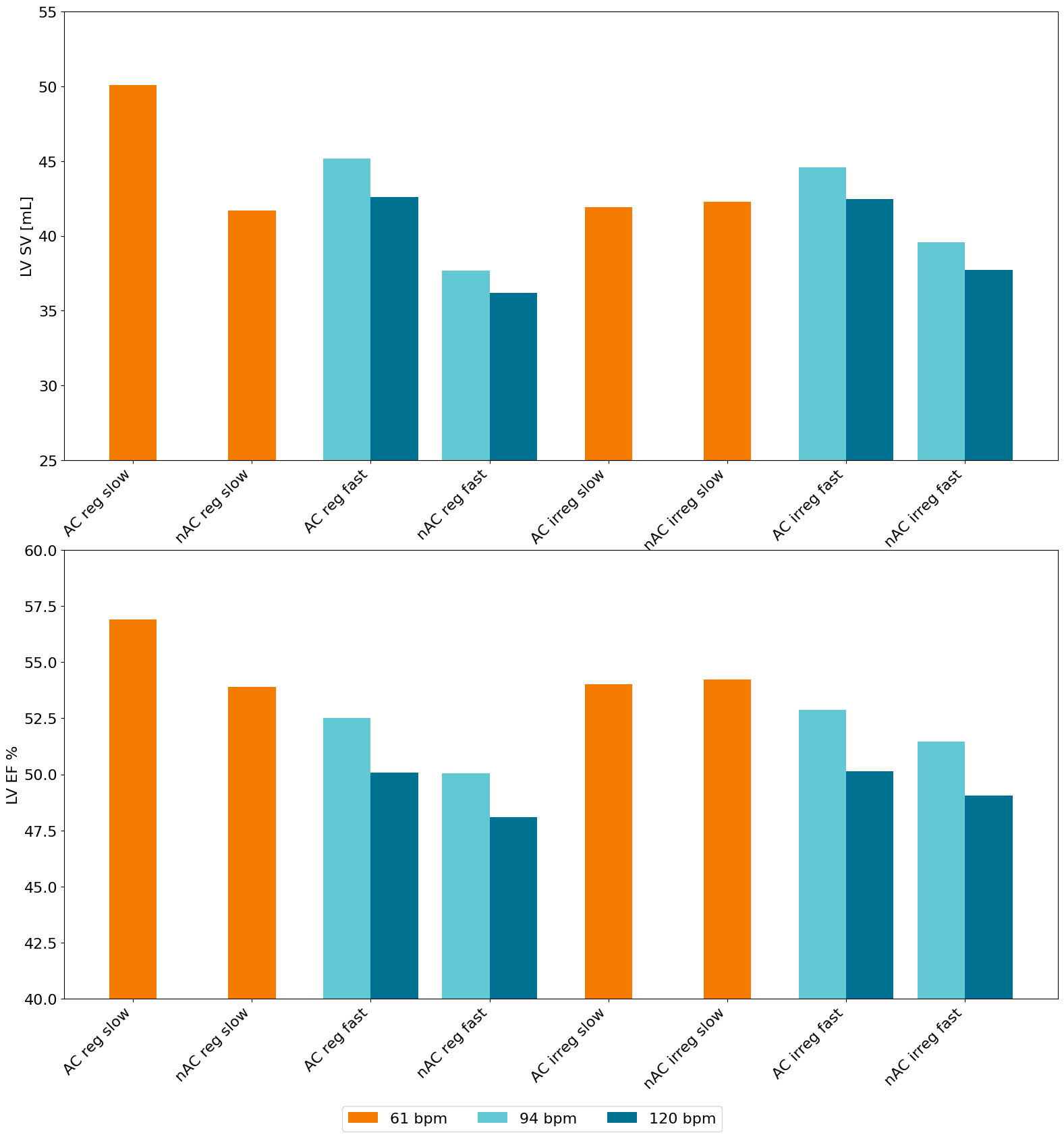
**

**Figure 13: Simulated LV SV and LV EF for one case in the cohort.** Simulations with higher heart rates have been included to ensure results are generalizable to AF patients experiencing faster ventricular rates.

**13: Effect of negative inotropic action of rate-control drugs**

This work focused on the primary hemodynamic effects of rate reduction but did not explicitly model drug-induced inotropic changes. For some rate control drugs, experimental data is available describing the inhibition of ion channels due to the drug.^42^ This data is presented as a concentration at which 50% channel block occurs (IC_50_) and a Hill coefficient of cooperativity (n). The percentage channel block can then be modeled as:

$$y=V_{max} . \frac{\left[ drug \right]^{n}}{\left[ {IC}_{50} \right]^{n}+ \left[ drug \right]^{n}}$$

where Vmax is 100.

Data in this form is available for the beta blocker sotalol (class II/III), where the free plasma concentration was assumed to be 14686.4 nM.^42^ Including the channel blocking effects of sotalol into our rate control simulations, we see no significant change in the LV EF. However, other common rate control drugs may have a negative inotropic effect, even if the data available to include this effect in the models is limited. The impact of including a mild reduction (-5% and -10%) decrease in contractility to reflect beta-blocker effects was therefore investigated. **Figure 14** shows the results of these simulations for one heart where the rate control simulation (slow, regular, no atrial contraction) serves as the baseline for comparison. LV SV and EF for these simulations are provided in **Table 6**.


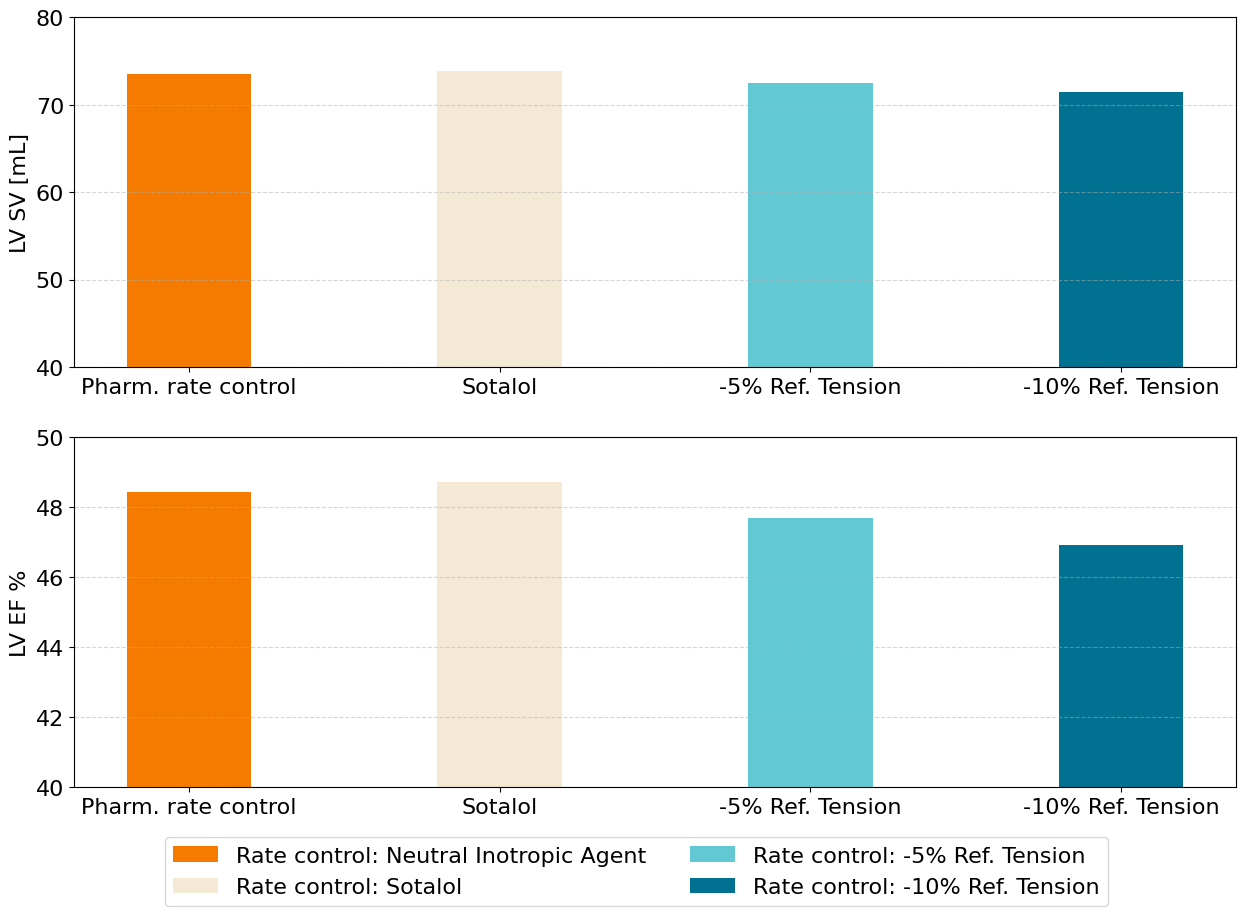


**Figure 14: Simulated LV SV and EF for one heart model in the cohort.** Sotalol was modelled using experimental channel block data and the case of a generic beta blocker causing a negative inotropic effect was also considered (5% and 10% decrease of the reference tension parameter in the cell model).

**Table 6: Simulated LV SV and EF for one heart model in the cohort.**

|  | **LV EF [%]** | **LV SV [mL]** |
| --- | --- | --- |
| **Neutral Inotropic Agent** | 48.4 | 73.5 |
| **Sotalol** | 48.7 | 73.9 |
| **5% decrease in ref. tension** | 47.7 | 72.5 |
| **10% decrease in ref. tension** | 46.9 | 71.4 |

Simulating rate control after sotalol administration results in a negligible change in LV EF (+0.3%) compared to simulating a slow, irregular rate with no atrial contraction (pharmacological rate control without accounting for possible negative inotropy). Similarly, simulation of a generic beta blocker with a 5% decrease in reference tension in the cell model led to a decrease of just 0.7% in LV EF compared to a simulation of pharmacological rate control with neutral inotropic effect. Therefore, the conclusion that rhythm control or paced rate control may be of limited benefit to patients for whom restoring atrial contraction in unlikely to be effective remains reasonable, assuming the negative inotropic effect of the selected beta blocker is mild.

Simulating a 10% decrease in reference tension to model a beta blocker with a stronger negative inotropic effect led to a decrease in LV EF of 1.5%, compared to pharmacological rate control with a neutral inotropic effect. While still a very moderate decrease, this suggests that there may be some limited benefit to a paced rate control or rhythm control strategy for patients who best tolerate rate control agents with a strong negative inotropic profile.

**References**

1. Longobardi S, Lewalle A, Coveney S, et al.: Predicting left ventricular contractile function via Gaussian process emulation in aortic-banded rats. Philosophical Transactions of the Royal Society A: Mathematical, Physical and Engineering Sciences 2020; 378:20190334.

2. Strocchi M, Longobardi S, Augustin CM, et al.: Cell to whole organ global sensitivity analysis on a four-chamber heart electromechanics model using Gaussian processes emulators. PLoS Comput Biol 2023; 19:e1011257.

3. Courtemanche M, Ramirez RJ, Nattel S: Ionic mechanisms underlying human atrial action potential properties: insights from a mathematical model. American Journal of Physiology-Heart and Circulatory Physiology 1998; 275:H301–H321.

4. Strocchi M, Longobardi S, Augustin CM, et al.: Cell to whole organ global sensitivity analysis on a four-chamber heart electromechanics model using Gaussian processes emulators. PLoS Comput Biol 2023; 19:e1011257.

5. Plank G, Loewe A, Neic A, et al.: The openCARP simulation environment for cardiac electrophysiology. Comput Methods Programs Biomed 2021; 208:106223.

6. Boyle PM, Zghaib T, Zahid S, et al.: Computationally guided personalized targeted ablation of persistent atrial fibrillation. Nat Biomed Eng 2019; 3:870–879.

7. Grand BL, Hatem S, Deroubaix E, Couetil J-P, Coraboeuf E: Depressed transient outward and calcium currents in dilated human atria. Cardiovasc Res 1994; 28:548–556.

8. Maier LS, Barckhausen P, Weisser J, Aleksic I, Baryalei M, Pieske B: Ca ^2+^ handling in isolated human atrial myocardium. American Journal of Physiology-Heart and Circulatory Physiology 2000; 279:H952–H958.

9. Gattoni S, Røe ÅT, Frisk M, Louch WE, Niederer SA, Smith NP: The calcium–frequency response in the rat ventricular myocyte: an experimental and modelling study. J Physiol 2016; 594:4193–4224.

10. Tahir H, Nguyen L, Sachdev S, et al.: Abstract P150: Age, Gender and Race Variations in QRS Duration in Patients With Atrial Fibrillation. Circulation 2019; 139.

11. Kreimer F, Aweimer A, Pflaumbaum A, Mügge A, Gotzmann M: Impact of P‐wave indices in prediction of atrial fibrillation—Insight from loop recorder analysis. Annals of Noninvasive Electrocardiology 2021; 26.

12. Strocchi M, Augustin CM, Gsell MAF, et al.: A publicly available virtual cohort of four-chamber heart meshes for cardiac electro-mechanics simulations. PLoS One [Internet] Public Library of Science, 2020; 15:e0235145-. Available from: https://doi.org/10.1371/journal.pone.0235145

13. Rodero C, Strocchi M, Marciniak M, et al.: Linking statistical shape models and simulated function in the healthy adult human heart. PLoS Comput Biol 2021; 17:e1008851.

14. Bayer J, Blake R, Plank G, Trayanova N: A Novel Rule-Based Algorithm for Assigning Myocardial Fiber Orientation to Computational Heart Models. Ann Biomed Eng 2012; 40:2243–2254.

15. Pfaller MR, Hörmann JM, Weigl M, et al.: The importance of the pericardium for cardiac biomechanics: from physiology to computational modeling. Biomech Model Mechanobiol [Internet] 2019; 18:503–529. Available from: https://doi.org/10.1007/s10237-018-1098-4

16. Labarthe S, Bayer J, Coudière Y, et al.: A bilayer model of human atria: mathematical background, construction, and assessment. EP Europace [Internet] 2014; 16:iv21–iv29. Available from: https://doi.org/10.1093/europace/euu256

17. Roney CH, Pashaei A, Meo M, et al.: Universal atrial coordinates applied to visualisation, registration and construction of patient specific meshes. Med Image Anal [Internet] 2019; 55:65–75. Available from: https://www.sciencedirect.com/science/article/pii/S1361841518308089

18. Neic A, Campos FO, Prassl AJ, et al.: Efficient computation of electrograms and ECGs in human whole heart simulations using a reaction-eikonal model. J Comput Phys [Internet] 2017; 346:191–211. Available from: https://www.sciencedirect.com/science/article/pii/S0021999117304655

19. Houthuizen P, Bracke FALE, van Gelder BM: Atrioventricular and interventricular delay optimization in cardiac resynchronization therapy: physiological principles and overview of available methods. Heart Fail Rev 2011; 16:263–276.

20. Gillette K, Gsell MAF, Bouyssier J, et al.: Automated Framework for the Inclusion of a His–Purkinje System in Cardiac Digital Twins of Ventricular Electrophysiology. Ann Biomed Eng 2021; 49:3143–3153.

21. Tomek J, Bueno-Orovio A, Rodriguez B: ToR-ORd-dynCl: an update of the ToR-ORd model of human ventricular cardiomyocyte with dynamic intracellular chloride. bioRxiv [Internet] 2020; :2020.06.01.127043. Available from: http://biorxiv.org/content/early/2020/06/01/2020.06.01.127043.abstract

22. Courtemanche M, Ramirez RJ, Nattel S: Ionic mechanisms underlying human atrial action potential properties: insights from a mathematical model. American Journal of Physiology-Heart and Circulatory Physiology 1998; 275:H301–H321.

23. Land S, Park-Holohan S-J, Smith NP, dos Remedios CG, Kentish JC, Niederer SA: A model of cardiac contraction based on novel measurements of tension development in human cardiomyocytes. J Mol Cell Cardiol 2017; 106:68–83.

24. Guccione JM, McCulloch AD, Waldman LK: Passive Material Properties of Intact Ventricular Myocardium Determined From a Cylindrical Model. J Biomech Eng 1991; 113:42–55.

25. Strocchi M, Gsell MAF, Augustin CM, et al.: Simulating ventricular systolic motion in a four-chamber heart model with spatially varying robin boundary conditions to model the effect of the pericardium. J Biomech [Internet] 2020; 101:109645. Available from: https://www.sciencedirect.com/science/article/pii/S002192902030052X

26. Ogden RW: Nearly isochoric elastic deformations: Application to rubberlike solids. J Mech Phys Solids 1978; 26:37–57.

27. Flory PJ: Thermodynamic relations for high elastic materials. Transactions of the Faraday Society 1961; 57:829.

28. Arts T, Delhaas T, Bovendeerd P, Verbeek X, Prinzen FW: Adaptation to mechanical load determines shape and properties of heart and circulation: the CircAdapt model. American Journal of Physiology-Heart and Circulatory Physiology 2005; 288:H1943–H1954.

29. Walmsley J, Arts T, Derval N, et al.: Fast Simulation of Mechanical Heterogeneity in the Electrically Asynchronous Heart Using the MultiPatch Module. PLoS Comput Biol 2015; 11:e1004284.

30. Vigmond EJ, Hughes M, Plank G, Leon LJ: Computational tools for modeling electrical activity in cardiac tissue. J Electrocardiol 2003; 36:69–74.

31. Augustin CM, Gsell MAF, Karabelas E, et al.: A computationally efficient physiologically comprehensive 3D–0D closed-loop model of the heart and circulation. Comput Methods Appl Mech Eng 2021; 386:114092.

32. Field ME, Wasmund SL, Page RL, Hamdan MH: Restoring Sinus Rhythm Improves Baroreflex Function in Patients With Persistent Atrial Fibrillation. J Am Heart Assoc 2016; 5.

33. Miyoshi M, Kondo H, Ishii Y, et al.: Baroreflex Sensitivity in Patients With Atrial Fibrillation. J Am Heart Assoc 2020; 9.

34. McEvoy JW, McCarthy CP, Bruno RM, et al.: 2024 ESC Guidelines for the management of elevated blood pressure and hypertension. Eur Heart J 2024; .

35. Saljoughian M: Hypotension: A Clinical Care Review. USPharm 2014; 39.

36. Heidenreich PA, Bozkurt B, Aguilar D, et al.: 2022 AHA/ACC/HFSA Guideline for the Management of Heart Failure: A Report of the American College of Cardiology/American Heart Association Joint Committee on Clinical Practice Guidelines. Circulation 2022; 145.

37. Clifford G, Liu C, Moody B, et al.: AF Classification from a Short Single Lead ECG Recording: the Physionet Computing in Cardiology Challenge 2017. 2017,.

38. Marwick TH: Ejection Fraction Pros and Cons: JACC State-of-the-Art Review. J Am Coll Cardiol 2018; 72:2360–2379.

39. Rawles JM: What is meant by a “controlled” ventricular rate in atrial fibrillation? Heart 1990; 63:157–161.

40. St. Pierre SR, Peirlinck M, Kuhl E: Sex Matters: A Comprehensive Comparison of Female and Male Hearts. Front Physiol 2022; 13.

41. Frank O: On the dynamics of cardiac muscle. Am Heart J 1959; 58:282–317.

42. Crumb WJ, Vicente J, Johannesen L, Strauss DG: An evaluation of 30 clinical drugs against the comprehensive in vitro proarrhythmia assay (CiPA) proposed ion channel panel. J Pharmacol Toxicol Methods 2016; 81:251–262.
